# The ELF3-regulated lncRNA UBE2CP3 is over-stabilized by RNA–RNA interactions and drives gastric cancer metastasis via miR-138-5p/ITGA2 axis

**DOI:** 10.1038/s41388-021-01948-6

**Published:** 2021-07-17

**Authors:** Dandan Li, Jiajun She, Xinhui Hu, Meixin Zhang, Ruonan Sun, Shanshan Qin

**Affiliations:** 1 Hubei Key Laboratory of Embryonic Stem Cell Research, School of Basic Medical Sciences, Hubei University of Medicine, Shiyan, Hubei P.R. China; 2 Laboratory of Tumor Biology, Academy of Bio-Medicine Research, Hubei University of Medicine, Shiyan, Hubei P.R. China

**Keywords:** Cancer prevention, Gastric cancer

## Abstract

LncRNAs play essential roles in tumorigenesis and tumor progression. Pseudogene UBE2CP3 is an antisense intronic lncRNA. However, the biological function of UBE2CP3 in gastric cancer (GC) remains unknown. In this study, we revealed that lncRNA UBE2CP3 was aberrantly upregulated in multiple independent gastric cancer cohorts, and its overexpression was clinically associated with poor prognosis in GC. UBE2CP3 was mainly located in cytoplasm and promoted migratory and invasive capacities of GC cells in vitro and in vivo. Mechanismly, a novel dysregulated ceRNA network UB2CP3/miR-138-5p/ITGA2 was identified in GC by transcriptome sequencing. Furthermore, rescue assay further confirmed that UBE2CP3 mainly promoted GC progression through miR-138-5p/ITGA2 axis. More importantly, our data proved that UBE2CP3/IGFBP7 could form an RNA duplex, thereby directly interacting with the ILF3 protein. In turn, this RNA-RNA interaction between IGFBP7 mRNA and UBE2CP3 mediated by ILF3 protein plays an essential role in protecting the mRNA stability of UBE2CP3. In addition, transcription factor ELF3 was identified to be a direct repressor of lncRNA UBE2CP3 in GC. Taken together, overexpression of UBE2CP3 promotes tumor progression via cascade amplification of ITGA2 upregulation in GC. Our finding has revealed that the dysregulation of UBE2CP3 is probably due to the downregulation of ELF3 and/or the overexpression of IGFBP7 mRNA in GC. Our findings reveal, for the first time, that UBE2CP3 plays crucial a role in GC progression by modulating miR-138-5p/ITGA2 axis, suggesting that UBE2CP3 may serve as a potential therapeutic target in GC.

## Introduction

Gastric cancer (GC) is one of the most common cancer worldwide with the 5th highest incidence rate in all cancers [1]. The 5-years overall survival rate of GC patients remains unsatisfactory to date due to patients were usually diagnosed at an advanced stage with limited therapeutic strategies [2, 3]. Although there are great advances in the treatment of gastric carcinogenesis, the molecular mechanisms underlying tumorigenesis and metastasis are still poorly understood.

Long non-coding RNAs (lncRNAs) are a class of ncRNA that greater than 200 nt in length, and usually have no potential capacity to encode protein. Thanks to the popularization of high-throughput sequencing technology, large number of lncRNAs, mRNAs and miRNAs have been found to be dysregulated in tumors [4, 5]. And these dysregulated functional RNAs are actually linked to each other according to the ceRNA hypothesis proposed by Pandolfi et al. in 2011 [6]. That is, the dysregulation of ceRNA network (lncRNA-miRNA-mRNA network), in fact, led to tumorigenesis and metastasis [7, 8]. Therefore, exploring and identifying new ceRNA networks in tumors will become a hot spot in the field of cancer research and treatment.

Pseudogenes transcripts actually are considered as a special type of lncRNAs [9]. Increasing evidence have shown that pseudogene lncRNAs were aberrantly dysregulated in cancers and played essential roles in tumorigenesis [10]. Recently, several studies regarding pseudogene lncRNAs in gastric cancer have been published. For examples, lncRNAs POU5F1B, a pseudogene of OCT4, has been proved to promote an aggressive phenotype in GC [11]. LncRNA DUXAP8/10, pseudogenes of DUXA, have been reported to play an oncogenic role in GC progression [12, 13]. LncRNA KRT19P3, pseudogene of KRT19, could suppresses proliferation and metastasis in GC [14]. However, the biological function of most dysregulated pseudogene lncRNAs in GC remains unclear.

In this study, we identified an aberrantly up-regulated pseudogene lncRNA (named UBE2CP3) in GC. Our data showed that the increased expression of lncRNA UBE2CP3 was clinically associated with poor prognosis of GC patients. A novel ceRNA network composed of UBE2CP3, miR-138 and ITGA2 was identified to be responsibility for the poor prognosis of GC patients. In addition, UBE2CP3 was negatively regulated by the transcription factor ELF3, but positively stabilized by IGFBP7 mRNA via interacting with ILF3 protein. To date, our work present here represents the first data on the precise function of lncRNA UBE2CP3 in gastric cancer.

## Results

### The subcellular localization, expression pattern and prognosis of different homologous UBE2C pseudogenes varies widely

**Fig. 1:**
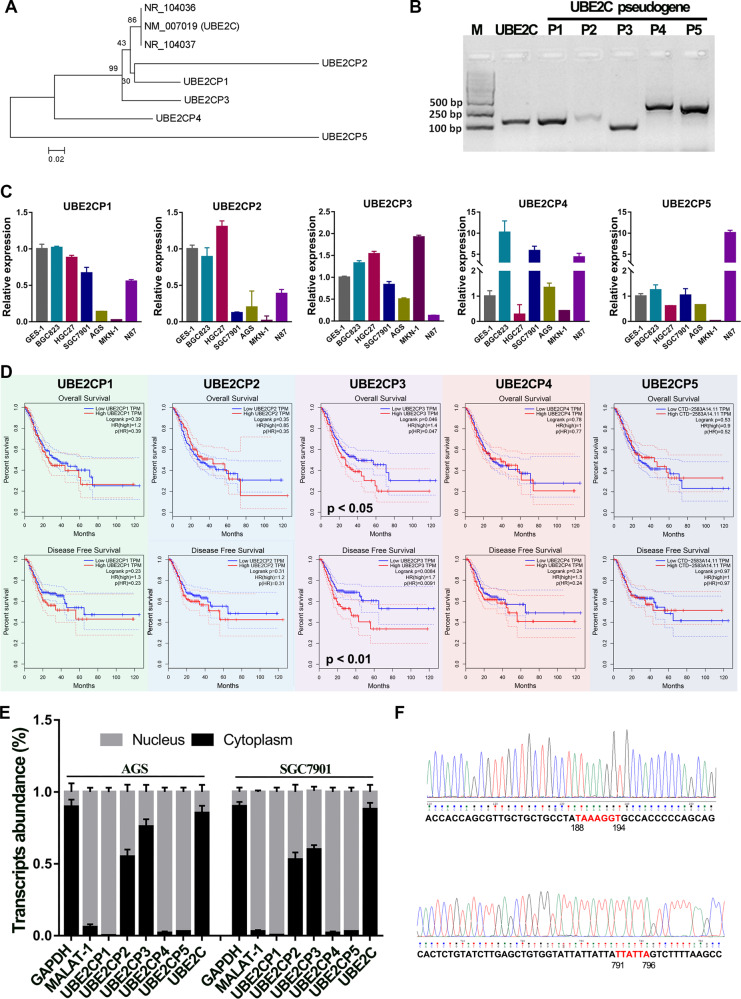
The expression profile, prognostic analysis and subcellular location of UBE2C pseudogenes in GC. **A** Phylogenetic analysis of 5 UBE2C pseudogenes and UBE2C using their human cDNA sequence. **B** The RT-PCR assay showed that all the UBE2C pseudogenes could be transcribed in SGC7901 cell line. **C** The expression profile of 5 UBE2C pseudogenes in different GC cell lines. ATCB is used as an internal reference gene. **D** The Kaplan–Meier OVS (up panel) and DFS (bottom panel) analysis of 5 UBE2C pseudogenes in GC patients from TCGA cohort using the GEPIA web tool. The GC patients in TCGA cohort were divided into two groups (High expression and Low expression) according to the expression level of UBE2C pseudogenes. The *P* values were estimated using log-rank test. **E** The subcellular distribution of transcripts of 5 UBE2C pseudogenes in GC cell lines. **F** Sanger sequencing results of UBE2CP3 cDNA obtained from SGC7901 cell line.

Five pseudogenes of ubiquitin-conjugating enzyme 2 C (UBE2C) were identified by BLAST searches of human genome using UBE2C nucleoid sequence, including UBE2CP1, UBE2CP2, UBE2CP3, UBE2CP4, and UBE2CP5. Phylogenetic analysis shows that UBE2CP1, UBE2CP2, and UBE2CP3 have higher sequence similarity with UBE2C compared with UBE2CP4 and UBE2CP5 (Figs. 1A, S1). In order to analyze the expression patterns of these pseudogenes in GC cells, we designed specific primers that can specifically distinguish these pseudogenes with highly similar sequences (Fig. 1B). The expression analysis showed that UBE2C pseudogenes possessed differential expression in different GC cell lines (Fig. 1C). UBE2CP3 was highly expressed in MKN-1, HGC27 and BGC823 GC cell lines, but lowly expressed in GC cell line N87. UBE2CP1 expression was almost declined in all the GC cell lines. UBE2CP5 was mainly expressed in N87. It’s worth mentioning that the most abundant UBE2C pseudogenes transcripts in GC cell lines were UBE2CP1, UBE2CP3, and UBE2CP5, according to the Ct value of each UBE2C pseudogene in the qPCR assay. This result may imply that UBE2CP1, UBE2CP3, and UBE2CP5 were more likely to play a role in the progression of gastric cancer.

On the other hand, the survival analysis of GC patients from TCGA cohort showed that among the 5 UBE2C pseudogenes, only UBE2CP3’s expression was significantly related to the prognosis (OVS + DFS) of GC patients (Fig. 1D). Besides, the nuclear/cytoplasmic RNA separation assay showed that UBE2CP1, UBE2CP4, and UBE2CP5 were mainly located in nucleus of GC cell lines, while UBE2CP2 and UBE2CP3 were distributed in cytoplasm and nucleus of GC cell lines (Fig. 1E). Given UBE2CP3 overexpression could predict a poor prognosis in GC, we decided to further explore the biological role of UBE2CP3 in GC. Thus, the full length of UBE2CP3 was cloned by RACE method. After sequencing, we found that the UBE2CP3 sequence provided by NCBI has two deletions (Fig. 1F), compared with the correct UBE2CP3 sequence (Table S1). In addition, we also used genomic DNA as a template to amplify and sequence the UBE2CP3 gene (data not shown). The results showed that the two deletion was not due to RNA editing since the sequence of UBE2CP3 products using cDNA and the sequence of UBE2CP3 products using gDNA are same.

### LncRNA UBE2CP3 is aberrantly upregulated and correlated with poor prognosis in GC

**Fig. 2:**
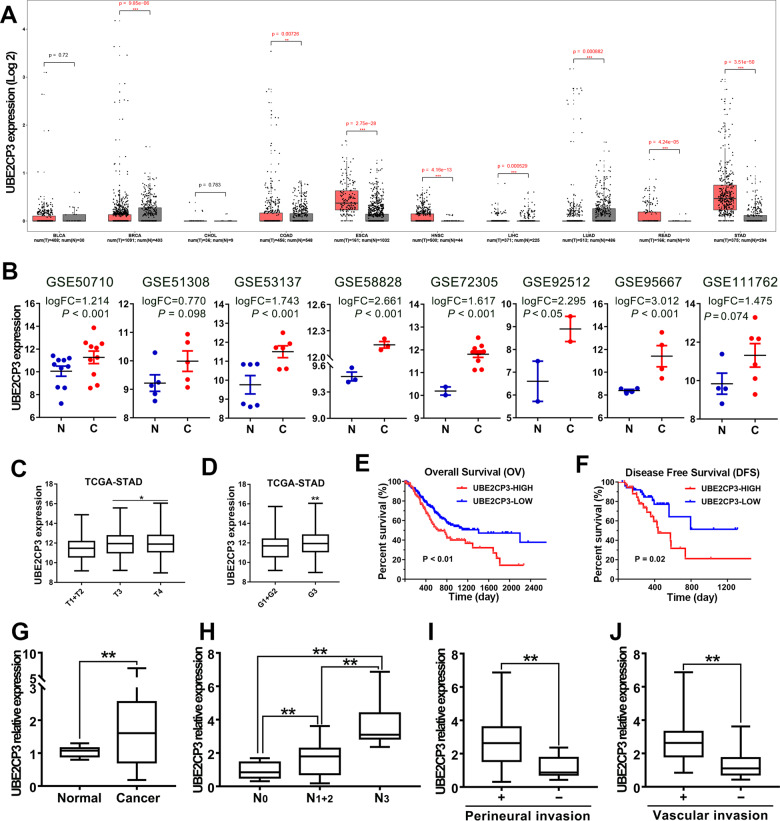
The overexpression of UBE2CP3 was clinically associated with poor prognosis of GC patients. **A** The expression of UBE2CP3 in different tumor cohort that contained corresponding normal tissues was obtained by pan-cancer analysis of the transcriptome data from TCGA. The *P* values were estimated using two-tailed *t* test. **B** The expression of UBE2CP3 in different independent GC cohort was obtained by analysis of the available lncRNA microarray data. The *P* values were estimated using two-tailed *t* test. **C** The expression level of UBE2CP3 in different T-stages of GC tissues from TCGA cohort. The *P* values were estimated using Mann–Whitney nonparametric test. **D** Difference in expression levels of UBE2CP3 in GC tissues with different degrees of differentiation. The *P* values were estimated using Mann–Whitney nonparametric test. **E**, **F** GC patients with relative higher expression of UBE2CP3 possessed a shorter overall survival time and disease-free survival time. The *P* values were estimated using log-rank test. **G** The UBE2CP3 expression was examined in 30 paired GC samples by qRT-PCR assay. The *P* values were estimated using two-tailed *t* test. **H** The expression level of UBE2CP3 is positively correlated with lymph node metastasis. The *P* values were estimated using two-tailed paired *t* test. **I**, **J** GC patients with high expression of UBE2CP3 are more prone to perineural invasion and vascular invasion. The *P* values were estimated using two-tailed *t* test. **P < 0.01.

To explore the biological function of UBE2CP3 in cancer, we first conducted the pan-cancer analysis of UBE2CP3 expression in the different types of TCGA cancer cohorts containing the corresponding normal tissues. It is worth noting that many types of cancer have to be excluded because they have no normal tissue in TCGA. Our results confirmed that UBE2CP3 was overexpressed in most types of tumors, including breast cancer (BRCA), colon cancer (COAD), esophageal cancer (ESCA), head and neck squamous cell carcinoma (HNSC), rectum adenocarcinoma (READ), and stomach cancer (STAD), suggesting UBE2CP3 may function important roles in tumor progression (Fig. 2A). To further verify the upregulation of UBE2CP3 in GC, we also examined the UBE2CP3 expression profile in human gastric cancer tissues and normal stomach tissues by analysis of the available expression data in GEO dataset. Eight GC microarray gene profiling datasets were downloaded from NCBI website because they belonged to platform GPL15314 and contained the expression data of UBE2CP3. After re-annotation and analysis of the differential expression data, we verified that lncRNA UBE2CP3 was aberrantly upregulated in all eight microarray datasets (Fig. 2B). Since UBE2CP3 was confirmed to be overexpressed in GC, we further analyzed the correlation between UBE2CP3 expression and clinicopathological and prognosis of GC patients. It’s found that the overexpression of UBE2CP3 was positively correlated with the degree of malignancy of gastric tumors (Fig. 2C). Similarly, the UBE2CP3 expression in poorly differentiated gastric cancer tissues was higher than that in moderately or highly differentiated gastric cancer tissues (Fig. 2D, *p* < 0.0001). To evaluate the association between UBE2CP3 expression and the prognosis of patients with gastric cancer, 373 patients were assigned to the high UBE2CP3 expression groups (*n* = 152) and the low UBE2CP3 expression group (*n* = 221). The survival analysis revealed that patients possessed higher expression level of UBE2CP3 had both a shorter overall survival (OV) time and a shorter disease-free (DFS) time than those with lower UBE2CP3 expression level (Fig. 2E, F).

On the other hand, we also investigated the expression level of UBE2CP3 by qRT-PCR analysis in the GC cohort (*n* = 30) collected by ourselves. The results also showed that lncRNA UBE2CP3 transcripts were expressed at higher levels in GC tissue than non-tumor tissue from the same donor (*P* < 0.001; Fig. 2G). In addition, the expression of UBE2CP3 was positively correlated with lymph node metastasis of gastric cancer (Fig. 2H). Compared with patients with low expression of UBE2CP3, patients with high expression of UBE2CP3 are more prone to perineural invasion and vascular invasion (Fig. 2I, J). These results strongly suggested that UBE2CP3 might play an oncogenic role in GC.

### UBE2CP3 positively regulates EMT signalling and promotes GC progression in vivo and in vitro

**Fig. 3:**
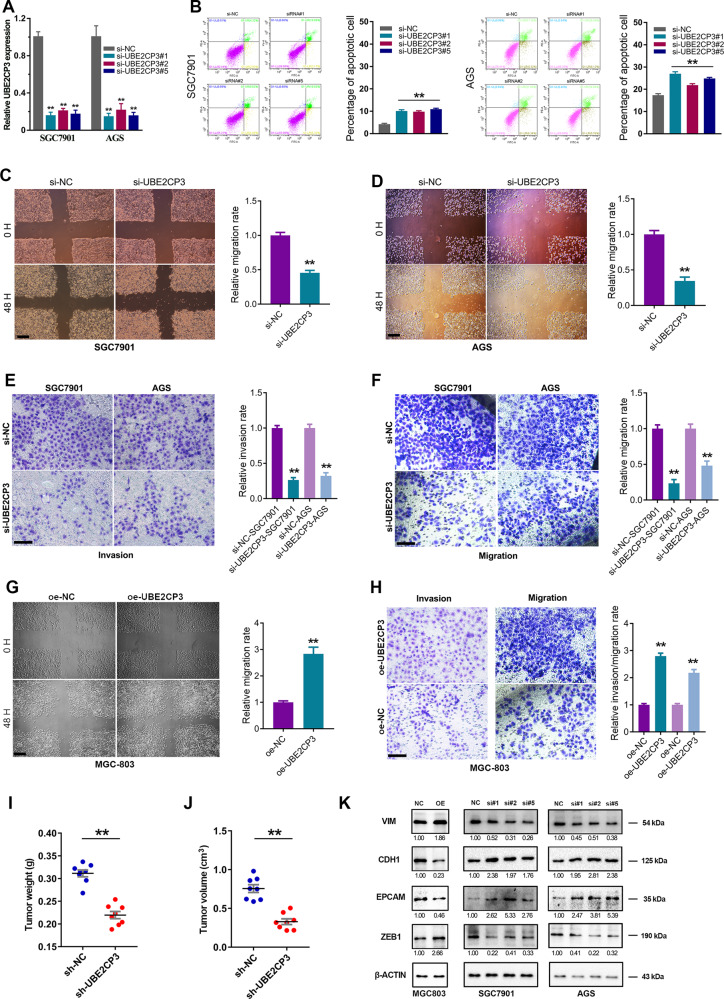
UBE2CP3 promotes GC cell growth and metastasis in vitro and in vivo. **A** The knockdown efficiency of UBE2CP3 in GC cell lines was verified by qRT-PCR assay. The *P* values were estimated using one-way ANOVA test. **B** Knockdown of UBE2CP3 significantly promoted cell apoptosis rate of GC cell lines. **C**, **D** The effects of UBE2CP3 knockdown on GC cells migration were assessed by wound healing assay in GC cell lines. **E**, **F** The effects of UBE2CP3 knockdown on GC cells migration and invasion were assessed by transwell assay in GC cell lines. **G**, **H** The effects of UBE2CP3 overexpression on the migration and invasion of GC cells were assessed by wound healing assay and transwell assay. **I**, **J** The weight and volume of harvested transplanted tumors were measured. **K** The effect of UBE2CP3 overexpression and depletion on the expression of EMT biomarkers was determined in different GC cell lines by western blotting. The scale bars in wound healing assay and transwell assay represent 100 μm and 25 μm, respectively. NC negative control. The *P* values were estimated using two-tailed *t* test. **P < 0.01.

To verify the cancer-promoting effect of UBE2CP3 in GC, loss-of-function studies regarding on UBE2CP3 were conducted. Five different siRNAs of UBE2CP3 in total were designed to avoid off-target effect. Three of them were proved to be able to efficiently knock down UBE2CP3 expression in GC cell lines (Fig. 3A). Therefore, these three effective siRNAs were subsequently used in the following experiments. To investigate the effect of UBE2CP3 knockdown on GC cells proliferation, MTT assays were performed in both SGC7901 and AGS cells. The result showed that the growth ability of SGC7901 and AGS cells transfected with UBE2CP3 siRNAs were significantly hindered compared with control cells (Figs. S2A). Besides, the cell cycle assay and the cell apoptosis assay showed that silencing UBE2CP3 could remarkably affect the cell cycle distribution and increase the apoptosis rate of GC cells (Fig. 3B, Fig. S2B, C).

In addition, we further investigated the effect of UBE2CP3 expression on GC cells migration and invasion by performing wound healing assays and transwell assays. The results revealed that knockdown of UBE2CP3 significantly inhibited the gastric cancer cells migration and invasion, while overexpression of UBE2CP3 in turn remarkably promotes GC cell migration and invasion (Fig. 3C–H). These findings together indicated that lncRNA UBE2CP3 played an oncogenic role in GC progression and metastasis in vitro.

To explore whether the level of lncRNA UBE2CP3 expression affects tumorigenesis in vivo, the stable UBE2CP3-depleted SGC7901 cells were used in a nude mice xenograft model. After collected the transplant tumors, there was a dramatic decrease in tumor volume and weight in the sh-UBE2CP3 group compared with controls (Fig. 3I, J). These results suggest that the level of lncRNA UBE2CP3 expression is significantly associated with the in vivo proliferation capacity of gastric cancer cells.

Clinicopathological analysis showed that UBE2CP3 expression is closely associated with the metastasis and prognosis of GC patients. Besides, UBE2CP3 could promotes GC cell migration and invasion. Given EMT signalling pathway is critical for tumor metastasis, we also examined the expression level of certain EMT biomarker proteins by western blotting. The data showed that knockdown of UBE2CP3 decreased the expression of the mesenchymal biomarkers (ZEB1 and VIM) but increased the expression of epithelial biomarkers (CDH1 and EPCAM) in GC cell lines (Fig. 3K). That means, UBE2CP3 may promote GC metastasis through activation of EMT signalling pathway.

### LncRNA UBE2CP3 positively regulate ITGA2 expression by sequestrating miR-138

**Fig. 4:**
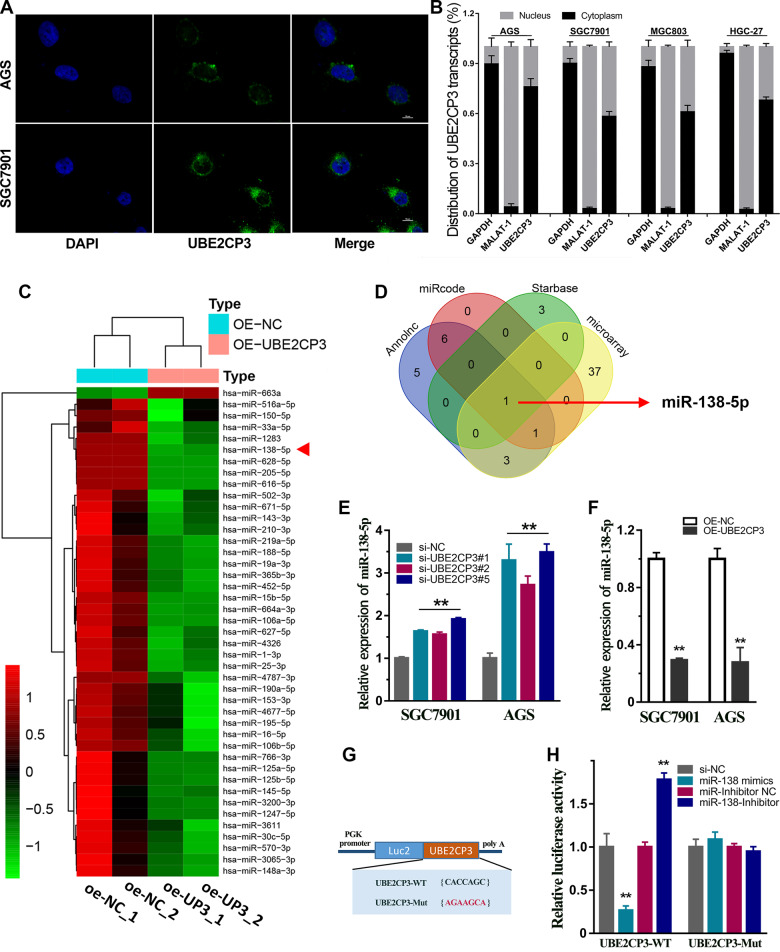
UBE2CP3 functioned as a sponge of miR-138-5p in GC. **A** The subcellular localization of UBE2CP3 was identified by RNA FISH assay in SGC7901 and AGS cell lines. Bar, 10 µm. **B** The UBE2CP3 transcripts were located in the cytoplasm and nucleus of GC cell lines. The Nuclear-localized lncRNA MALAT1 and cytoplasmic-localized GAPDH mRNA served as controls. **C** The heat map reveals the differential expressed miRNAs after exogenous UBE2CP3 overexpression in SGC7901 cell line. **D** The Venn diagram indicated UBE2CP3 might be a sponge of miR-138-5p. **E**, **F** The expression level of miR-138-5p was detected after knockdown or overexpression of UBE2CP3 in GC cell lines. The *P* values were estimated using one-way ANOVA test. **P < 0.01. **G** The schematic plot of dual-luciferase reporter vectors for wild-type UBE2CP3 and mutant UBE2CP3. **H** miR-138-5p directly binds to the UBE2CP3 sequence to regulate the Luc2 expression. The *P* values were estimated using two-tailed *t* test. **P < 0.01.

It is well known that the biological function of lncRNA is closely related to its subcellular location [15]. Therefore, RNA Fish assays were conducted in two different GC cell lines. The results revealed that the transcripts of UBE2CP3 were located in the nucleus and cytoplasm of GC cell lines (Fig. 4A). Besides, the nuclear-cytoplasmic RNA fractionation assay followed by qRT-PCR showed that about 50–70% of UBE2CP3 transcripts were in the cytoplasm of all four GC cell lines (Fig. 4B).

Cytoplasmic lncRNA can function as a ceRNA to regulate the expression of miRNAs that can be adsorbed by it [16]. Given this, microRNA sequencing studies was performed in the UBE2CP3-overexpressing SGC7901 cells (Fig. S2D) to screen the differentially expressed miRNAs in the GC cells overexpressing UBE2CP3. The high-throughput analysis revealed that a total of 42 miRNAs significantly decreased their expression after overexpression of UBE2CP3 (Fig. 4C). On the other hand, the possible microRNA (miRNA)-response elements (MRE) in the lncRNA UBE2CP3 sequence were predicted by three different bioinformatics algorithms, including miRCode, Annolnc and Starbase v3.0 (Fig. S3). It is worth noting that the miRNA-UBE2CP3 interactions predicted by Starbase v3.0 is supported by the Ago2 CLIP-seq data (Fig. S3C). After intersecting the bioinformatics prediction results obtained by different algorithms with the down-regulated miRNA in the chip, only miR-138-5p meets all the requirements (Fig. 4D). It implied that lncRNA UBE2CP3 could function as an ceRNA by sponging miR-138-5p. To verify this possibility, we detected the miR-138-5p expression after overexpression of UBE2CP3 or knockdown of UBE2CP3. The results showed that UBE2CP3 negatively regulated the expression of miR-138-5p in GC (Fig. 4E, F). Besides, the dual-luciferase reporter assay showed that the negative regulation of UBE2CP3 on the expression of miR-138-5p greatly weakened after mutation of miR-138-5p seed sequence binding site (Fig. 4G, H). Taken together, pseudogene lncRNA UBE2CP3 could exert a ceRNA effect through sponging miR-138-5p in GC.

**Fig. 5:**
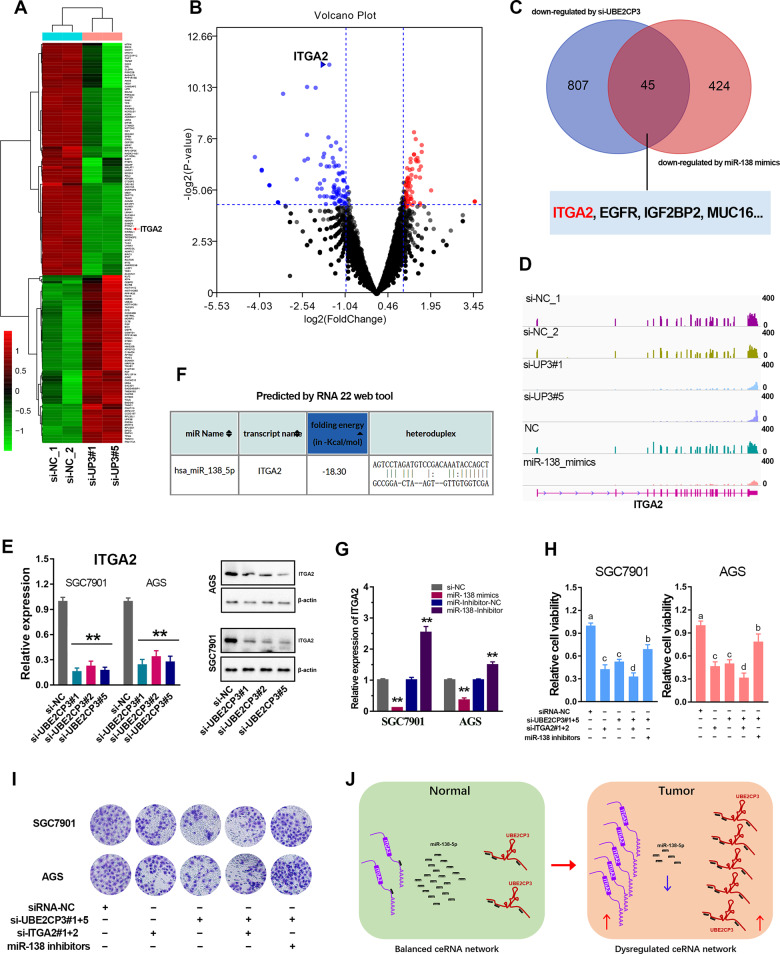
UBE2CP3 positively regulates GC progression through miR-138-5p/ITGA2 axis. **A** The heat map reveals the differential expressed genes after UBE2CP3 knockdown. **B** The differential expressed genes (|Log2FC | >1, *p* < 0.05) after UBE2CP3 depletion were shown in the volcano plot. ITGA2 is one of the most down-regulated genes (Log2FC = −2.17, *p* < 0.0001) by UBE2CP3 depletion. **C** The genes that can be simultaneously down-regulated by miR-138-5p mimics (Log2FC < −0.9) and UBE2CP3 depletion (Log2FC < −1). **D** The transcripts abundance of ITGA2 after UBE2CP3 knockdown and miR-138-5p overexpression. The ordinate represents counts per million (CPM) value (0-400). **E** The expression of ITGA2 was determined after knockdown of UBE2CP3 by qRT-PCR assay and western blotting. The *P* values were estimated using one-way ANOVA test. **P < 0.01. **F** The binding sites and folding energy of miR-138-5p on the ITGA2 mRNA was analyzed by RNA 22 web tool. **G** The expression of ITGA2 was determined after transfected with miR-138-5p mimics and inhibitors in GC cell lines. **H** CCK-8 assays for SGC7901 and AGS cells transfected with siRNAs targeting UBE2CP3 and ITGA2, miR-138-5p inhibitors and controls. **I** Transwell invasion assays for SGC7901 and AGS cells transfected with siRNAs targeting UBE2CP3 or ITGA2, miR-138-5p inhibitors and controls. **J** The deduced schematic diagram of the cancer-promoting mechanism derived from the ceRNA network UBE2CP3/miR-138/ITGA2. The *P* values were estimated using two-tailed *t* test. **P < 0.01.

Based on the ceRNA hypothesis proposed by Pandolfi [17], we further speculated that UBE2CP3 may regulate targeted mRNAs by sequestration of shared miR-138-5p. Therefore, RNA sequencing analysis was conducted in the UBE2CP3-depletion GC cells. The differentially expressed mRNAs (|log2FC | >1) after knockdown of UBE2CP3 were shown in Fig. 5A. The volcano plot showed that ITGA2 (log2FC = −2.17, *p* < 0.001) was the most significantly down-regulated gene among those genes downregulated by UBE2CP3 (Fig. 5B, Table S2). On the other way, we also conducted the RNA sequencing analysis in the GC cells transfecting miR-138-5p mimics and the negative control miRNA. After intersecting the downregulated genes (log2FC < −1) by UBE2CP3-depletion and the downregulated genes (log2FC < −1) by miR-138-5p mimics, ~45 genes were listed in the Venn plot. It’s worth noting that ITGA2 was one of them (Fig. 5C). The RNA-seq analysis showed that the abundance of ITGA2 transcripts was indeed declined in the UBE2CP3-depletion or miR-138-5p overexpression GC cells (Fig. 5D). Besides, the positive regulation of UBE2CP3 on the ITGA2 expression in GC cell lines was further verified by qPCR and western blotting assays (Figs. 5E, S4).

To verify whether ITGA2 could be a direct target gene of miR-138-5p, we predicted the miR-138-5p binding sites in the ITGA2 transcripts using RNA22 web tool (Fig. 5F). The results showed that miR-138-5p could directly bind on the ITGA2 transcripts (folding energy = −18.3 Kcal/mol). In addition, ITGA2 expression could negatively regulated by miR-138-5p mimics, but positively regulated by miR-138-5p inhibitors (Fig. 5G). These results strongly suggested that there is an ceRNA network composed by UBE2CP3, miR-138-5p and ITGA2 in GC.

Furthermore, the rescue assays showed that the promoting effect of UBE2CP-depletion on the growth of GC cell lines can be restored by miR-138 inhibitors but can be further enhanced by exogenous siRNAs targeting ITGA2 (Fig. 5H). Besides, the transwell assays also showed that the promoting effect of UBE2CP-depletion on the invasion of GC cell lines can be counterweighed by miR-138 inhibitors but can be further strengthened by extra knockdown of ITGA2 (Fig. 5I). These data strongly suggested that UBE2CP3 promotes GC progression through miR-138-5p/ITGA2 axis.

Taken together, we proposed a possible model that UBE2CP3 may work in GC. In the normal gastric cells, the expression of UBE2CP3, miR-138-5p and ITGA2 were balanced. In the tumor cells, the dysregulation of UBE2CP3 will lead to the amplified dysregulation of miR-138-5p and ITGA2. Finally, this cascaded dysregulation of ceRNA network strongly promotes the malignant progression of gastric cancer (Fig. 5J).

### UBE2CP3 transcripts could be stabilized by IGFBP7 mRNA via interaction with ILF3 protein

**Fig. 6:**
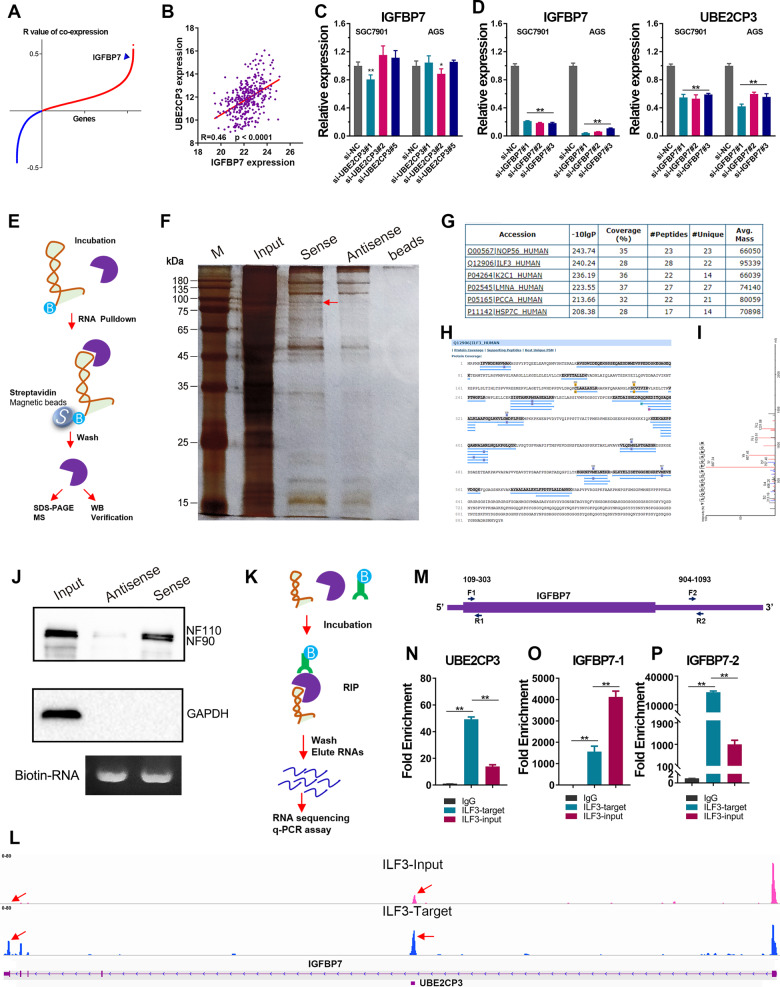
UBE2CP3 mRNA was stabilized by IGFBP7 mRNA by forming a duplex RNA to interact with ILF3 protein. **A** The correlation (*R* value) of co-expression between each gene and UBE2CP3. **B** The IGFBP7 mRNA was highly co-expressed with UBE2CP3 mRNA. The *P* value was estimated using Pearson correlation test. **C** IGFBP7 expression was examined after silencing UBE2CP3 expression in GC cell lines by qRT-PCR assay. The *P* values were estimated using one-way ANOVA test. **P < 0.01. **D** The knockdown efficiency of IGFBP7 in GC cell lines was verified by qRT-PCR assay (left panel). UBE2CP3 expression was examined after silencing IGFBP7 expression in GC cell lines by qRT-PCR assay (right panel). The *P* values were estimated using one-way ANOVA test. **P < 0.01. **E** Schematic workflow of the RNA pull-down assay for identification of UBE2CP3 binding proteins. The sense (S) and anti-sense (AS) of UBE2CP3 RNA were biotinylated, refolded, and incubated with SGC7901 cell lysates. **F** Silver staining of the gel after SDS-PAGE of the potential UBE2CP3 binding proteins. Specific bands were excised and analyzed through mass spectrometry. The red arrow indicates gel band unique to UBE2CP3. The unique gel band was sent for mass spectrometry (MS) protein detection. **G** The top six protein hits according to the MS analysis of band labeled with a red arrow. **H** The sequence coverage of RNA binding proteins (ILF3) in the MS analysis was shown in the plot. **I** Single peptide-based protein identification of ILF3 protein. **J** Validation of UBE2CP3 binding proteins that obtained by RNA pulldown-MS method using western blotting. **K** Schematic workflow of the RNA Immunoprecipitation (RIP) assay for identification of UBE2CP3 binding proteins. **L** The transcripts abundances of UBE2CP3 and IGFBP7 were analyzed based on RIP-seq data. The ordinate represents counts per million (CPM) value (0–80). **M** The schematic diagram of the primer position of IGFBP7. **N**–**P** The transcript abundance of UBE2CP3, IGFBP7-5’UTR and IGFBP7-3’UTR was detected in the different group (IgG, anti-ILF3, input) of RIP assay by qRT-PCR. The *P* values were estimated using one-way ANOVA test. **P < 0.01.

UBE2CP3 was an intronic antisense lncRNA of IGFBP7 gene. Interestingly, we observed a high co-expression between UBE2CP3 and IGFBP7 in GC (Fig. 6A, B). In order to figure out whether this co-expression is caused by the mutual regulatory relationship between UBE2CP3 and IGFBP7, we knocked down of UBE2CP3 and IGFBP7 in GC cell lines, respectively. The results showed that knockdown of UBE2CP3 had no obvious effects on IGFBP7 expression in GC cell lines (Fig. 6C). However, the depletion of IGFBP7 caused a significant decline in the expression of UBE2CP3 in GC cell lines (Fig. 6D). Thus, we considered the co-expression of IGFBP7 and UBE2CP3 was due to IGFBP7 positively regulates UBE2CP3 expression.

LncRNAs may perform biological functions by interacting with proteins. Thus, we also performed RNA pull-down assay to screen possible proteins UBE2CP3 may interacted (Fig. 6E). The SDS-PAGE of UBE2CP3 pulldown assay showed that the protein pulled down by the sense UBE2CP3 and the antisense UBE2CP3 had a distinct band around 90 K Daltons (Fig. 6F). Thus, we cut off the gel in this area and performed mass spectrometry (MS) analysis. According to the MS results, the probable proteins in this band were shown in Table S3. The protein with highest matching coverage were ILF3, suggested UBE2CP3 could bind to ILF3 protein (Fig. 6G–I). Besides, the western blotting assay further confirmed that UBE2CP3 could interacted with ILF3 protein (Fig. 6J).

How did IGFBP7 regulate UBE2CP3 expression? ILF3 was a double-stranded RNA binding protein. Thus, we analyzed the interaction between IGFBP7 mRNA and UBE2CP3 mRNA using the IntaRNA online web tool (Fig. S5). The prediction results showed that the 627–679 nt region of UBE2CP3 could interact with 3’UTR of IGFBP7 mRNA (1006–1060 nt). To further verify the prediction results, we also conducted RNA immunoprecipitation-seq assay using ILF3 antibodies (Fig. 6K). As shown in the Fig. 6L, the UBE2CP3 transcripts pulled down by ILF3 antibody was extremely higher than the IgG control group, strongly suggested that ILF3 could bind to UBE2CP3 mRNA. Moreover, compared with the input group, we also noted an obvious peak located at the 3’ UTR region of IGFBP7 mRNA in the ILF3-target group, suggested that ILF3 could bind to the 3’ UTR region of IGFBP7 mRNA. To further confirmed the RIP-seq results, RIP-qPCR assays were conducted. The abundance of UBE2CP3 transcripts and the 3’UTR of IGFBP7 mRNA pulled down by ILF3 antibody was extremely higher than the IgG control group and the input group. It indicated that the results obtained from RIP-qPCR data and RIP-seq data are highly consistent. It has been reported that ILF3 can regulate transcripts stability by interacting with target mRNA. Therefore, IGFBP7 mRNA may interact with ILF3 by forming double-stranded RNA with UBE2CP3, thereby protecting the stability of UBE2CP3 transcripts.

Correspondingly, an obvious decrease in the expression of ITGA2 was observed by analysis of the RNA-seq data of IGFBP7 depletion (Fig. S6A, B). The qPCR analysis also showed that the ITGA2 expression was significantly decreased in the IGFBP7-depletion GC cell lines (Fig. S5C). Besides, expression analysis found that the expression level of IGFBP7 in tumors was significantly higher than that in normal tissues (Fig. S6D). Survival analysis also showed that GC patients with high expression of IGFBP7 possessed a relatively poor prognosis (OVS + DFS) in TCGA stomach cancer cohort and the GSE62254 stomach cancer cohort (Fig. S6E, F), suggested that IGFBP7 functioned as an oncogene in GC. Given UBE2CP3 was positively regulated by IGFBP7, and IGFBP7 depletion significantly decreased the expression of ITGA2, which was turned out to be a downstream target of UBE2CP3, we speculated that IGFBP7 might regulate GC progression through UBE2CP3/ITGA2 axis.

### LncRNA UBE2CP3 is negatively regulated by transcription factor ELF3 in GC

**Fig. 7:**
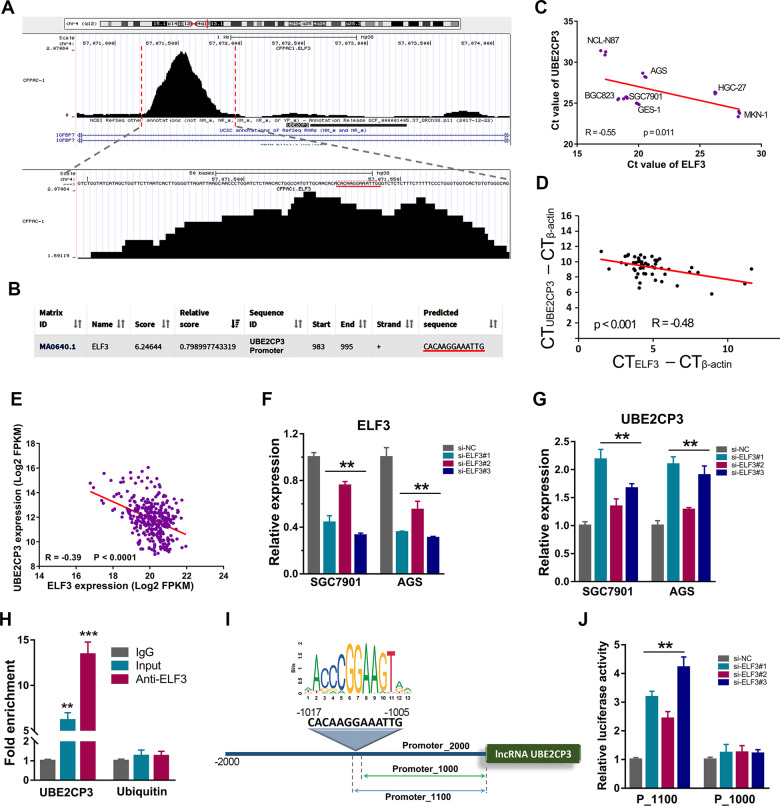
UBE2CP3 was negatively regulated by transcription factor ELF3 in GC. **A** The CHIP-seq data of transcription factor ELF3 was visualized through the Cistrome website. The center of the peak located at UBE2CP3 promoter contains a sequence of CACAAGGAAATTG. **B** The 2000 bp-length of UBE2CP3 promoter was analyzed by JASPAR web tool to predicted possible ELF3 binding sites. **C** Co-expression analysis of UBE2CP3 and ELF3 in GC cell lines using Ct value. The Ct value (*n* = 3) of UBE2CP3 and ELF3 in each GC cell line was obtained from qRT-PCR assay. The *P* value was estimated using Pearson correlation test. **D** The co-expression of UBE2CP3 and ELF3 was analyzed by qRT-PCR analysis in 30 paired of GC tissues. The *P* value was estimated using Pearson correlation test. **E** The co-expression of UBE2CP3 and ELF3 (log2FPKM value) was analyzed in the GC tissues from TCGA cohort. The *P* value was estimated using Pearson correlation test. **F** The knockdown efficiency of ELF3 in GC cell lines was verified by qRT-PCR assay. The *P* values were estimated using one-way ANOVA test. **P < 0.01. **G** UBE2CP3 expression was examined after silencing ELF3 expression in GC cell lines by qRT-PCR assay. The *P* values were estimated using one-way ANOVA test. **P < 0.01. **H** The CHIP-qPCR analysis in SGC7901 cells showed that the abundance of promoter fragment of UBE2CP3 pulled down by anti-ELF3 was much higher than that of the control IgG group. The *P* values were estimated using two-tailed *t* test. **P < 0.01. **I** The schematic diagram of truncated UBE2CP3 promoter (none ELF3 binding site) and the UBE2CP3 promoter containing ELF3 binding site. **J** GC cell line SGC7901 was co-transfected with siRNAs targeting ELF3 and luciferase reporter vector. After 48 h of incubation, luciferase activity was measured. The *P* values were estimated using two-tailed *t* test. **P < 0.01.

To identify which transcription factor could directly regulate UBE2CP3 expression in GC, we first screened all possible transcription factors that can bind to the UBE2CP3 promoter region by analysis of the available chip-seq data using the online web tool Cistrome (Table S4). Transcription factor E74 Like ETS Transcription Factor 3 (ELF3/ESE-1) was one of them according to the analysis result. As shown in the bottom of Fig. 7A, an obvious ELF3 peak was observed in the promoter region of UBE2CP3. After further analysis, we found that the center of the peak contains a sequence “CACAAGGAAATTG”. This sequence is also the possible ELF3 binding site in the UBE2CP3 promoter predicted by JASPAR Web server (Fig. 7B). It suggested that lncRNA UBE2CP3 might be transcriptionally regulated by ELF3.

Our previous published study showed that ELF3 was highly expressed in N87 cells, but lowly expressed in the HGC-27 cells [18]. However, on the contrary, UBE2CP3 was lowly expressed in N87 cells, but highly expressed in the HGC-27 cells. We used the Ct value of ELF3 and UBE2CP3 to analyze their expression correlation in GC cell lines and tissues. The results showed that the expression of ELF3 was negatively correlated to the UBE2CP3 expression in GC cell lines and GC tissues (Fig. 7C, D). Besides, the negative correlation between UBE2CP3 and ELF3 could be also observed in GC tissues from TCGA cohort (Fig. 7E). In order to confirm whether ELF3 can regulate UBE2CP3 expression, we examined the UBE2CP3 expression level after knockdown of ELF3 in GC cell lines. The results showed that knockdown expression of ELF3 significantly increased UBE2CP3 expression (Fig. 7F, G). Moreover, the result of CHIP assay showed that ELF3 could directly bind on the UBE2CP3 promoters in GC cells (Fig. 7H). In addition, the dual-luciferase reporter assay showed that the negative regulation of ELF3 on the UBE2CP3 expression could be counteracted when the ELF3 binding site was truncated (Fig. 7I, J). These results together indicated that transcription factor ELF3 negatively regulated UBE2CP3 expression via directly binding on the UBE2CP3 promoter.

**Fig. 8:**
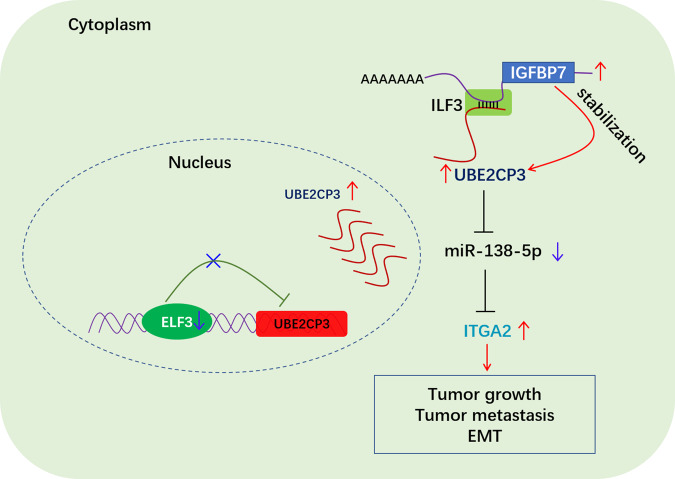
Hypothetical model for the function of pseudogene lncRNA UBE2CP3 in GC. Pseudogene lncRNA UBE2CP3 acts as a sponge for miR-138-5p and promotes GC progression through regulating miR-138-5p/ITGA2 axis. In addition, UBE2CP3 was negatively regulated by transcription factor ELF3, but positively regulated by IGFBP7 mRNA. ELF3 negatively regulates UBE2CP3 expression by directly binding to UBE2CP3 promoter. IGFBP7 mRNA forms duplex RNA with UBE2CP3 mRNA to stabilize UBE2CP3 mRNA via interacting with ILF3 protein.

Taken together, we mapped the working model of UBE2CP3’s cancer-promoting mechanism (Fig. 8). According to the working model, pseudogene lncRNA UBE2CP3, miR-138-5p and ITGA2 mRNA together form a ceRNA network. However, the downregulation of ELF3 and/or the increased level of IGFBP7 mRNA result in the abnormal overexpression of UBE2CP3 in GC. Subsequently, the aberrant ceRNA network UBE2CP3-miR-138-5p-ITGA2 contributes to the cascade amplification of ITGA2 upregulation in GC. Ultimately, the overexpression of ITGA2 led to the occurrence of EMT and metastasis of GC.

## Discussion

Non-coding RNA (ncRNA) constitute the majority of the human transcriptome, including a class of short ncRNAs (such as miRNAs and piRNAs), lncRNAs (such as lincRNAs, antisense RNAs, pseudogenes…) and circular RNAs [19]. Mountain evidence show that ncRNAs are functional and related to many diseases, especially cancer [20]. These ncRNAs usually form a ceRNA network with coding genes through competitive regulatory interaction, thereby modulating the expression of critical coding genes to regulate cancer progression [21]. Thus, the abnormal expression of any network component may derail complex regulatory circuits and ultimately lead to the development and progression of cancer [22]. So far, the biological function of many coding genes in GC or other tumors has been revealed. However, the cause of the dysregulation of many coding genes in cancers remains unclear. Therefore, clarifying the ceRNA regulatory network of these genes can help us better understand the mechanism of tumor metastasis.

In this study, we found the subcellular localization, expression pattern and prognosis of 5 homologous UBE2C pseudogenes vary widely in GC. Among the 5 UBE2C pseudogenes, only UBE2CP3, a lncRNA mainly located in cytoplasm, predicted a poor prognosis in GC. The significant overexpression of lncRNA UBE2CP3 was observed in multiple independent GC cohorts. Besides, UBE2CP3 overexpression was positively correlated with advanced TNM stage and metastasis in GC. Consistently, Gain-and loss-of-function studies in GC cell lines revealed that UBE2CP3 positively regulates GC cell proliferation, migration and invasion, and negatively regulates GC cell apoptosis. Similarly, subcutaneously implanted tumor model in nude mice showed that UBE2CP3 depletion significantly represses GC tumor growth in vivo. These results strongly implied that UBE2CP3 functioned an oncogenic role by promoting GC metastasis.

As a cytoplasmic lncRNA, UBE2CP3 may regulate GC metastasis through ceRNA mechanism [15]. Therefore, RNA-seq studies regarding UBE2CP3 overexpression/knockdown were performed to identify the corresponding downstream miRNAs and coding genes. Based on the transcriptome sequencing data, a novel ceRNA network “UBE2CP3/miR-138-5p/ITGA2” was identified in GC. Previous studies showed that integrin protein play essential roles in EMT process [23, 24]. As one of the important members of integrin, ITGA2 was also proved to be a potent inducer of EMT in tumors [25, 26]. Unanimously, our data also reveals that UBE2CP3 was also involved in promoting EMT signaling pathway in GC cell lines.

Rescue assay proved that UBE2CP3 promote GC metastasis mainly through miR-138-5p/ITGA2 axis. The ceRNA network composed of UBE2CP3, miR-138-5p and ITGA2 was dysregulated in GC. Increasing studies have shown that miR-138-5p plays a tumor suppressor effect in a variety of cancers [27, 28]. Pang et al. reported that miR-138-5p was downregulated in GC [29]. Recently, Dong et al. have reported that ITGA2 was upregulated and predicted poor prognosis in GC [29, 30]. In 2017, Dong et al. reported that ITGA2 depletion reduced the cell motility of GC cells through repressing EMT signaling pathway [31]. In 2018, Chuang et al. reported that blockade of ITGA2 by antibodies promoted cell apoptosis and inhibited cell migration in GC [32]. Subsequently, Ren et al. reported that ITGA2 plays an oncogenic role in pancreatic cancer, stomach cancer, liver cancer and breast cancer by activating the STAT3 pathway [33]. According to the ceRNA theory, the overexpression of UBE2CP3 would lead to a sharp increase in the expression of ITGA2 through competitive binding of miR-138-5p (Fig. 5J). In other words, it’s the dysregulation of the ceRNA network “UBE2CP3/miR-138/ITGA2” that drives the malignant progression and metastasis of gastric carcinoma.

In addition, with the help of CHIP assay and RNA pulldown assay, we identified two factors that were involved in regulating the expression of UBE2CP3. One is the transcription factor ELF3, the other is IGFBP7 mRNA. Our finding proved that ELF3 acted as a repressor of UBE2CP3 transcription. And IGFBP7 mRNA could maintain stabilization of lncRNA UBE2CP3 by interacting with ILF3 protein. The abnormal overexpression of UBE2CP3 or dysregulated ceRNA network (UBE2CP3/miR-138/ITGA2) may be due to the downregulation of ELF3 and/or the increased level of IGFBP7 mRNA in GC.

In conclusion, a novel ceRNA regulatory network (UBE2CP3/miR-138/ITGA2) was identified in GC. The oncogenic lncRNA UBE2CP3 mainly promotes GC progression and metastasis through miR-138-5p/ITGA2 axis (Fig. 8). Importantly, the ceRNA network was found to be dysregulated in GC due to the downregulation of ELF3 and the up-regulation of IGFBP7 mRNA. Our data highlights UBE2CP3 played essential roles in the modulation GC progression and metastasis, considering UBE2CP3 as a novel prognostic marker and therapeutic target in GC. To our knowledge, the results presented here represent the first data on the function of the lncRNA UBE2CP3 in human GC.

## Materials and methods

The Supplementary Materials and Methods document includes detailed procedures for: microarray and pan-cancer analysis [15]; selected cell lines, culture and transfection conditions; RNA sequencing of UBE2CP3 knockdown (GSE163813), microRNA sequencing studies of UBE2CP3 overexpression (GSE163814) and the RNA-seq of the transcripts pulled down by ILF3 antibodies (GSE163815); the Bioinformatic base on the RNA-seq data [34]; wound healing assay, cell invasion assay and cell proliferation assay; subcellular location of UBE2CP3; mouse xenograft model; chip assay regarding transcription factor ELF3 [18]; quantitative RT-PCR and related primers (Table S5); western blotting and information of selected antibodies (Table S6); RNA pulldown assay of lncRNA UBE2CP3; dual luciferase reporter assay; flow cytometry assay of UBE2CP3 knockdown in GC cell lines and statistical analysis.

## Supplementary information

Supplementary Materials and Methods

Legends of supplementary figures and Tables

Supplementary Figure S1

Supplementary Figure S2

Supplementary Figure S3

Supplementary Figure S4

Supplementary Figure S5

Supplementary Figure S6

Supplementary Table S1

Supplementary Table S2

Supplementary Table S3

Supplementary Table S4

Supplementary Table S5

Supplementary Table S6

## References

[1] Wang XH, Jiang ZH, Yang HM, Zhang Y, Xu LH (2021). Hypoxia-induced FOXO4/LDHA axis modulates gastric cancer cell glycolysis and progression. Clin Transl Med.

[2] Huang L, Jansen L, Balavarca Y, Verhoeven RHA, Ruurda JP, Van Eycken L (2020). Decreasing resection rates for nonmetastatic gastric cancer in Europe and the United States. Clin Transl Med.

[3] Yang Y, Zhang J, Chen Y, Xu R, Zhao Q, Guo W (2020). MUC4, MUC16, and TTN genes mutation correlated with prognosis, and predicted tumor mutation burden and immunotherapy efficacy in gastric cancer and pan-cancer. Clin Transl Med.

[4] Bala P, Singh AK, Kavadipula P, Kotapalli V, Sabarinathan R, Bashyam MD (2021). Exome sequencing identifies ARID2 as a novel tumor suppressor in early-onset sporadic rectal cancer. Oncogene.

[5] He D, Wang D, Lu P, Yang N, Xue Z, Zhu X (2021). Single-cell RNA sequencing reveals heterogeneous tumor and immune cell populations in early-stage lung adenocarcinomas harboring EGFR mutations. Oncogene.

[6] Shi X, Sun M, Liu H, Yao Y, Song Y (2013). Long non-coding RNAs: a new frontier in the study of human diseases. Cancer Lett.

[7] Pandolfi P (2012). The ceRNA hypothesis and the non-coding revolution in cancer research and therapy. Eur J Cancer.

[8] Lv Y, Wang Y, Song Y, Wang SS, Cheng KW, Zhang ZQ (2021). LncRNA PINK1-AS promotes Galphai1-driven gastric cancer tumorigenesis by sponging microRNA-200a. Oncogene.

[9] Lou W, Ding B, Fu P (2020). Pseudogene-derived lncRNAs and their miRNA sponging mechanism in human cancer. Front Cell Dev Biol.

[10] Liu Q, Hu X, Zhang X, Dai L, Duan X, Zhou C (2016). The TMSB4 pseudogene LncRNA functions as a competing endogenous RNA to promote cartilage degradation in human osteoarthritis. Mol Ther.

[11] Hayashi H, Arao T, Togashi Y, Kato H, Fujita Y, De Velasco MA (2015). The OCT4 pseudogene POU5F1B is amplified and promotes an aggressive phenotype in gastric cancer. Oncogene.

[12] Xu Y, Yu X, Wei C, Nie F, Huang M, Sun M (2018). Over-expression of oncigenic pesudogene DUXAP10 promotes cell proliferation and invasion by regulating LATS1 and beta-catenin in gastric cancer. J Exp Clin Cancer Res.

[13] Ma HW, Xie M, Sun M, Chen TY, Jin RR, Ma TS (2017). The pseudogene derived long noncoding RNA DUXAP8 promotes gastric cancer cell proliferation and migration via epigenetically silencing PLEKHO1 expression. Oncotarget.

[14] Zheng J, Zhang H, Ma R, Liu H, Gao P (2019). Long non-coding RNA KRT19P3 suppresses proliferation and metastasis through COPS7A-mediated NF-kappaB pathway in gastric cancer. Oncogene.

[15] Li D, Wang J, Zhang M, Hu X, She J, Qiu X (2020). LncRNA MAGI2-AS3 is regulated by BRD4 and promotes gastric cancer progression via maintaining ZEB1 overexpression by sponging miR-141/200a. Mol Ther Nucleic Acids.

[16] Karreth FA, Pandolfi PP (2013). ceRNA cross-talk in cancer: when ce-bling rivalries go awry. Cancer Disco.

[17] Tay Y, Rinn J, Pandolfi PP (2014). The multilayered complexity of ceRNA crosstalk and competition. Nature.

[18] Li D, Cheng P, Wang J, Qiu X, Zhang X, Xu L (2019). IRF6 is directly regulated by ZEB1 and ELF3, and predicts a favorable prognosis in gastric cancer. Front Oncol.

[19] Zhao S, Guan B, Mi Y, Shi D, Wei P, Gu Y (2021). LncRNA MIR17HG promotes colorectal cancer liver metastasis by mediating a glycolysis-associated positive feedback circuit. Oncogene.

[20] Wu LF, Mo XB, He JH, He P, Lu X, Deng HW (2021). Integrative lncRNA-mRNA co-expression network analysis identifies novel lncRNA E2F3-IT1 for rheumatoid arthritis. Clin Transl Med.

[21] Yan L, Liu G, Wu X (2021). The umbilical cord mesenchymal stem cell-derived exosomal lncRNA H19 improves osteochondral activity through miR-29b-3p/FoxO3 axis. Clin Transl Med.

[22] Chan JJ, Tay Y (2018). Noncoding RNA:RNA Regulatory Networks in Cancer. Int J Mol Sci.

[23] Canel M, Serrels A, Frame MC, Brunton VG (2013). E-cadherin-integrin crosstalk in cancer invasion and metastasis. J Cell Sci.

[24] Salemi Z, Azizi R, Fallahian F, Aghaei M (2021). Integrin alpha2beta1 inhibition attenuates prostate cancer cell proliferation by cell cycle arrest, promoting apoptosis and reducing epithelial-mesenchymal transition. J Cell Physiol.

[25] Gaballa R, Ali HEA, Mahmoud MO, Rhim JS, Ali HI, Salem HF (2020). Exosomes-mediated transfer of Itga2 promotes migration and invasion of prostate cancer cells by inducing epithelial-mesenchymal transition.. Cancers.

[26] Liu X, Liang Z, Gao K, Li H, Zhao G, Wang S (2016). MicroRNA-128 inhibits EMT of human osteosarcoma cells by directly targeting integrin alpha2. Tumour Biol.

[27] Zhang W, Liao K, Liu D (2020). MiR-138-5p inhibits the proliferation of gastric cancer cells by targeting DEK. Cancer Manag Res.

[28] Yeh M, Oh CS, Yoo JY, Kaur B, Lee TJ (2019). Pivotal role of microRNA-138 in human cancers. Am J Cancer Res.

[29] Pang L, Li B, Zheng B, Niu L, Ge L (2017). miR-138 inhibits gastric cancer growth by suppressing SOX4. Oncol Rep.

[30] Wang Q, Cao TY, Guo K, Zhou Y, Liu H, Pan YN (2020). Regulation of integrin subunit alpha 2 by miR-135b-5p modulates chemoresistance in gastric cancer. Front Oncol.

[31] Dong J, Wang R, Ren G, Li X, Wang J, Sun Y (2017). HMGA2-FOXL2 axis regulates metastases and epithelial-to-mesenchymal transition of chemoresistant gastric cancer. Clin Cancer Res.

[32] Chuang YC, Wu HY, Lin YL, Tzou SC, Chuang CH, Jian TY (2018). Blockade of ITGA2 induces apoptosis and inhibits cell migration in gastric cancer. Biol Proced Online.

[33] Ren D, Zhao J, Sun Y, Li D, Meng Z, Wang B (2019). Overexpressed ITGA2 promotes malignant tumor aggression by up-regulating PD-L1 expression through the activation of the STAT3 signaling pathway. J Exp Clin Cancer Res.

[34] Wang JJ, Zhang MX, Hu XH, She JJ, Sun RN, Qin SS (2021). miRNA-194 predicts favorable prognosis in gastric cancer and inhibits gastric cancer cell growth by targeting CCND1. Febs Open Bio.

