# Correction: The ELF3-regulated lncRNA UBE2CP3 is over-stabilized by RNA–RNA interactions and drives gastric cancer metastasis via miR-138-5p/ITGA2 axis

**DOI:** 10.1038/s41388-023-02698-3

**Published:** 2023-04-28

**Authors:** Dandan Li, Jiajun She, Xinhui Hu, Meixin Zhang, Ruonan Sun, Shanshan Qin

**Affiliations:** 1 Hubei Key Laboratory of Embryonic Stem Cell Research, School of Basic Medical Sciences, Hubei University of Medicine, Shiyan, Hubei P.R. China; 2 Laboratory of Tumor Biology, Academy of Bio-Medicine Research, Hubei University of Medicine, Shiyan, Hubei P.R. China

**Keywords:** Cancer prevention, Gastric cancer

Correction to: *Oncogene* 10.1038/s41388-021-01948-6, published online 17 July 2021

Following the publication of this article, the authors noted incorrect transwell images in Fig. 5i. A corrected version of Fig. 5i is given below. The description of the results and the figure legend remain unchanged, and the authors confirm that conclusions of this article are not affected by this correction. The authors apologize for any inconvenience this may have caused for readers.
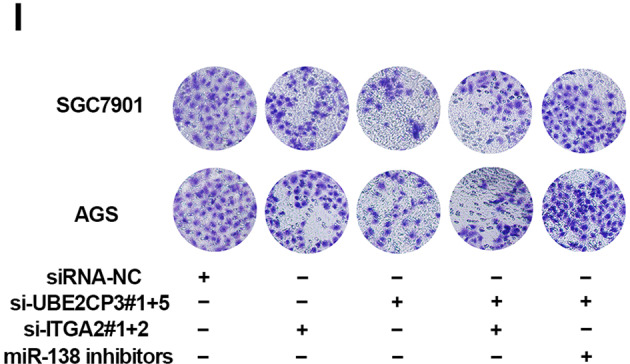


The original article has been corrected.

