# Supplementary material for: The ELF3-regulated lncRNA UBE2CP3 is over-stabilized by RNA–RNA interactions and drives gastric cancer metastasis via miR-138-5p/ITGA2 axis
Source: Oncogene. 2021 Jul 17;40(35):5403–15. doi: 10.1038/s41388-021-01948-6 (PMC8413130; doi:10.1038/s41388-021-01948-6)
Supplement: Supplementary file 1 — Supplementary Materials and Methods [file 41388_2021_1948_MOESM1_ESM.docx]

**Materials and methods**

**Microarray data analysis and pan-cancer analysis in TCGA.**

ArrayStar Human LncRNA Microarray V2.0 (GPL15314 platform) is designed for the global profiling of human LncRNAs and protein-coding transcripts. All public lncRNA microarray datasets (GSE50710, GSE51308, GSE53137, GSE58828, GSE72305, GSE93512, GSE95667 and GSE111762) used in this study were downloaded from the Gene Expression Omnibus (GEO) in the NCBI web server. The probe ID of lncRNA UBE2CP3 is ASHG19A3A025558. The survival analysis of UBE2C pseudogenes were analyzed by using GEPIA web server. RNA-Seq data of 407 gastric cancer samples and the correlated clinical information of 443 gastric cancer samples were downloaded from The Cancer Genome Atlas (TCGA). Expression level of per gene was calculated from log2 of its upper quartile FPKM (FPKM-UQ) value. The survival analysis of pan-cancer was conducted using the KM-plot web tool.

**Cell transfection**

Human gastric cancer cell lines AGS and HGC-27 were purchased from GeneChem (Shanghai, China). The human gastric cancer cell lines (BGC823, SGC7901, NCL-N87, MKN-1 and MGC803) and the normal gastric cell line GES-1 were purchased from the Shanghai Cell Bank of Chinese Academy of Sciences. The siRNAs were designed and synthesized by Genepharma (Shanghai, China). Briefly, GC cell lines were seeded into 6-well plates and grown overnight. The next day, when the cell plating density reached 20%-30%, GC cells were transfected with siRNAs (final concentration, 50 nM) by Lipofectamine 2000 (Invitrogen) according to the manufacturer’s instructions. At the indicated time points, the cells were harvested for mRNA and protein analysis as well as for other assays. The sequence of related siRNAs in this study was listed in the Table S1.

**Clinical GC samples**

Human primary gastric cancer samples were obtained from 30 patients who underwent surgical resection at Taihe hospital. The study protocol was approved by the Human Research Ethics Committee of Hubei University of Medicine (2018-TH-035). The procedures are in accordance with the Helsinki Decaration of 1975. Written informed consent was obtained from all patients. Tissue samples were immediately frozen in liquid nitrogen after resection and stored at − 80 °C until use. All samples were pathologically confirmed.

**RNA sequencing**

After 48h transfected with targeted siRNAs and corresponding negative control siRNAs, total RNA of GC cells was extracted to perform RNA sequencing. A total amount of 1.5 µg RNA per sample was used as input material for the RNA sample preparations. The whole step of library construction and sequencing was performed at Shanghai Lifegenes Technology Co., Ltd. The RNA-seq data was uploaded on the GEO section of NCBI web server. The gene expression omnibus accession number is GSE163813.

**MicroRNA seq analysis**

UBE2CP3 gene expression is effectively overexpressed in SGC7901 cells using pcDNA3.1 plasmid. 36 hours after transfection, the total RNA samples in SGC7901 cells were extracted. After confirming UBE2CP3 overexpression, the RNA samples were sent to BGI company (Wuhan, China) for microRNA purification and miRNA sequencing analysis. The normalized expression level of each microRNA was calculated by TPM value and Log2FC (UBE2CP3/NC) of TPM was calculated to estimate between-group differences. For each microRNA, the difference between negative control and the matched targeted samples was set to be significant if the fold change > 1. The microRNA-seq data was uploaded on the GEO section of NCBI web server. The gene expression omnibus accession number is GSE163814.

**Wound healing assay, Cell invasion assay** **and Cell proliferation assay**

For wound healing assay, a sterile 100 µl pipette tip was used to longitudinally scratch a constant-diameter stripe in the confluent monolayer. The medium and cell debris were aspirated away and replaced with 2 ml of fresh medium. Photographs were taken at 0 and 48 hours after wounding. For statistical analysis, ten randomly selected fields along each wound were marked, and the area of the wound was measured, and the average was calculated as the wound area of this wound.

For transwell assay, the upper chamber was placed into a 24-well plate containing 500 μL of medium containing 20% FBS. A cell suspension (200 μL) was added to the upper chamber of the Transwell module and incubated for 16 hours at 37 ^o^C in 5% CO_2_. The Transwell module was then washed twice with PBS and fixed with pre-chilled methanol for 10 minutes at -20 ^o^C. The upper chamber of the Transwell module was washed twice with PBS. The cells remaining on the top surface of the upper chamber were removed with a wet cotton swab. The upper chamber was then washed 3 times with PBS and air dried at the inverted position. The chamber membrane was stained with a 0.1% crystal violet staining solution at 500 μL per well for 30 minutes at 37 ^o^C, washed 3 times, and air dried.

For cell proliferation assays, cells were reseeded in 96-well plates at 2,000 cells/well in a final volume of 100 μL and cultured for 4 days. The GC cell proliferation activities were determined with CCK-8 assay every 24 hours. Subsequently, 10 μL of CCK-8 solution (Biosharp, China) were added into each well and incubated for 2 hours. Optical density was measured at a wavelength of 490 nm by an automatic microplate reader (Bio Tek, USA). Triplicate wells were assayed for each experiment, and three independent experiments were performed. Data were expressed as the OD490 mean ± S.D.

**Mouse xenograft model**

Four-week-old female BALB/c nude mice were purchased from the Laboratory Animal Center of Hubei University of Medicine and maintained in a temperature-controlled (21 °C) and light-controlled pathogen-free animal facility with free access to food and water. The study protocol was approved by the Experimental Animal Research Ethics Committee of Hubei University of Medicine (2019-056). All animals were treated in accordance with guidelines of the Committee on Animals of the Hubei University of Medicine. 5 × 10^6^ of UBE2CP3-depletion SGC7901 cells were injected into subcutaneous tissue of female BALB/c nude mice (8 mice per group). After 28 days, all the mice were sacrificed, and the tumors were collected for weighing and volume measurement. The tumor volume was calculated using the following formula: volume = length × (width)^2^/2.

**Nuclear and cytoplasmic RNA isolation**

GC cells were harvested for isolation of cytoplasm and nuclear using the nuclear/cytoplasmic separation kit (BB-36021-2, BestBio, China) according to the manufacturer’s instructions. The obtained cytoplasmic components and cytoplasmic components were added to 500 ul of Trizol, and then extracted RNA using Trizol reagent (Invitrogen, USA) according to the manufacturer’s instructions. For Quantitative RT-PCR, 1 ug of cytoplasm RNA and 1ug of nuclear RNA were reversed to obtain cDNA using the PrimeScript ^TM^ RT reagent Kit (Perfect Real Time, Takara). Relative fold changes of gene expression were calculated using the comparative ΔC_t_ method. The primers for quantitation of GAPDH and lncRNA MALAT-1 transcripts level were list in Table S1.

**RNA Fish assay**

Briefly, GC cell lines were seeded and fixed with 4% paraformaldehyde. The next day, when the cell plating density reached 50%-70%, GC cells were treated with 0.5% Triton followed by pre-hybridization. Overnight hybridization was performed with a 10 mM probe concentration. The RNA FISH kit was purchased from Ribo Bio (Guangzhou). The experiment was performed according to the manufacturer’s instructions. The 5’FAM-UBE2CP3 probes were designed and synthesized by Sangon Biotech (Shanghai). Images were taken with a confocal microscope (Zeiss).

**RNA isolation and Quantitative RT-PCR**

Total RNA was extracted using Trizol reagent (Invitrogen, USA). Reverse transcription was performed to obtain cDNA by using the PrimeScript TM RT reagent Kit (Perfect Real Time, Takara). The qPCR protocol was using One Step TB Green PrimeScript TM RT-PCR Kit II (Takara) according to the manufacturer’s instructions. The qPCR analysis was conducted on Bio-Rad CFX Manager 3.1 real-time PCR system. All the primers synthesized by Wcgene Biotech (Shanghai, China). RNU6B (U6), ACTB and GAPDH were used as internal controls. Each gene was run in triplicate. Relative fold changes of gene expression were calculated using the comparative ΔΔCt method. All primers were synthesized by Wcgene Biotech (Shanghai, China). The sequence of related primers in this study was listed in the Table S1.

**Chromatin immunoprecipitation assay**

Chromatin immunoprecipitation (CHIP) assays were performed using CHIP Assay Kit ([56383S](http://www.cst-c.com.cn/products/56383.html), Cell Signal Technology, USA) according to the manufacturer’s protocol as we previously described. Briefly, the SGC7901 cell line were collected and fixed for 10 min at 37 with 1% formaldehyde, followed in sequence with SDS lysis and DNA shearing, protein and DNA immunoprecipitation, cross-linked DNA reversal and DNA purification, and finally the immunoprecipitated DNA fragments were detected by PCR assays. The normal rabbit IgG was used as the negative control. The primers for CHIP was listed in Table S1.

**Western blot assay**

Gastric cancer cells were lysed in RIPA buffer added 1 mM PMSF. Approximately 100 μg of total protein was electrophoresed through 10% SDS polyacrylamide gels and were then transferred to a PVDF membrane (Millipone). After blocking with 5% skimmed milk at 4 °C for 1h, the membrane was incubated with primary antibody at 4°C overnights. The blots were then washed and incubated with horseradish peroxidase (HRP)-conjugated secondary antibody (1: 10000, Earthox) for 1.5 h at room temperature. Detection was performed by using a SuperLumia ECL HRP Substrate Kit (Abbkine) and visualized using a Bio-Rad Imaging System (USA). The average gray scale density value of each band was determined by digital analysis of image using ImageJ software. The relative expression value of each band was obtained by making the gray value of NC (negative control) band as 1. The detail information of antibodies used in this study was listed in Table S2.

**RNA Immunoprecipitation followed by RNA sequencing (RIP-seq)**

After crosslinking with 0.5% formaldehyde for 10 min at room temperature, cells were harvested and lysed in RIP lysis buffer with RNasin (1000 U/ml), DNase I (50 U/ml) and protease inhibitor cocktail. After the genomic DNA was digested, lysates were further subjected to sonication. Supernatants cleared by centrifugation were incubated with the anti-ILF3 antibody (Proteintech, China) or IgG overnight at 4 °C. Protein A/G beads were added for a further 4 h incubation at room temperature. After the beads were washed with wash buffer, immunocomplexes of proteins and RNAs were de-crosslinked at 95 °C for 15 min. The immunoprecipitated RNAs were then purified for RNA sequencing and qRT-PCR analysis.

For RNA sequencing, RNA samples were fragmented into fragments by RNA fragmentation buffer. First strand cDNA was synthesized using random hexamer primer and M-MuLV Reverse Transcriptase. Second strand cDNA synthesis was subsequently performed using DNA Polymerase I and RNase H. Remaining overhangs were converted into blunt ends via exonuclease/polymerase activities. After adenylation of 3’ ends of DNA fragments, NEBNext Adaptor with hairpin loop structure were ligated to prepare for hybridization. In order to select cDNA fragments of preferentially 250~300 bp in length, the library fragments were purified with AMPure XP system (Beckman Coulter, Beverly, USA). Then 3 µl USER Enzyme (NEB, USA) was used with size-selected, adaptor-ligated cDNA at 37℃ for 15 min followed by 5 min at 95℃ before PCR. Then PCR was performed with Phusion High-Fidelity DNA polymerase, Universal PCR primers and Index (X) Primer. At last, PCR products were purified (AMPure XP system) and library quality was assessed on the Agilent Bioanalyzer 2100 system. The RIP-seq data was uploaded on the GEO section of NCBI web server. The gene expression omnibus accession number is GSE163815.

**RNA pull-down assay**

Briefly, full length of UBE2CP3 sequences were cloned into pGEM-T Easy (Promega). In vitro transcription was carried out and RNA was purified and labeled with Biotin at 3′ end. Cells were harvested and resuspended in freshly prepared lysis buffer supplemented with 50 U/mL RNase inhibitor (Takara) and a protease/phosphatase inhibitor cocktail (Roche). Sense and antisense RNAs were captured on magnetic beads (Pierce) and were incubated with cell lysates in protein-RNA-binding buffer (Thermo Scientific) overnight at 4 °C with agitation. RNA-binding protein complexes were washed five times with ice-cold wash buffer and were boiled in SDS lysis buffer for western blot assay and mass spectrometry (MS) analysis.

**Dual luciferase reporter assay**

The wildtype and truncated UBE2CP3 promoters were amplified by PCR using genome DNA from SGC7901 cells. The truncated UBE2CP3 promoter (none ELF3 binding site) and the wildtype UBE2CP3 promoter (contain putative ELF3 binding site) were ligated into the pEZX-FR01-dual luciferase reporter vector (GeneCopoeia, USA). The mutated UBE2CP3 cDNA sequence was constructed from wildtype promoters by using seamless cloning to replace CACCAGC with AGAAGCA (Yeasen Biotech, China). Then, the wildtype UBE2CP3 cDNA sequence and the mutant UBE2CP3 cDNA sequence were ligated into the pEZX-FR02-dual luciferase reporter vector (GeneCopoeia, USA). SGC7901 cells were seeded into 12-well-tissue plates 24h before transfection, and then co-transfected with 5ng siRNA (siRNAs targeting ELF3 or miR-138 mimics/inhibitors) and 1mg corresponding plasmid using the Lipofectamine 2000 Reagent (Invitrogen), according to the manufacturer’s instructions. After another 48h, cells were assayed using the Dual-Luciferase reporter assay system kit (GeneCopoeia, USA). All experiments were performed in triplicate and data were pooled from three independent experiments.

**Flow cytometry assay**

After 48h transfected with siRNAs and corresponding negative control siRNAs, SGC7901 and AGS cells were collected and performed cell cycle assay and cell apoptosis assay in accordance with the manufacture's protocol (BB-4104, BestBio, CHINA). Flow cytometry assays was performed on the CytoFLEX machine (Beckman, USA). The cell cycle and cell apoptosis distribution were quantified using the CytExpert software.

**Statistical analysis**

For gene expression analysis of different subtypes of GC, the P values were estimated using Mann–Whitney nonparametric test. Survival curves were calculated using the Kaplan–Meier method, and differences between the curves were analyzed using the log-rank test. Pearson correlation analysis was used for the correlation test of the two groups of data. All the rest of the experiments were used unpaired t-test or one-way ANOVA test. *t*-test were used for the comparison of composition ratio between groups. All experiments were repeated at least three times unless stated otherwise. All tests with p values less than 0.05 considered statistically significant.
