# Supplementary material for: The ELF3-regulated lncRNA UBE2CP3 is over-stabilized by RNA–RNA interactions and drives gastric cancer metastasis via miR-138-5p/ITGA2 axis
Source: Oncogene. 2021 Jul 17;40(35):5403–15. doi: 10.1038/s41388-021-01948-6 (PMC8413130; doi:10.1038/s41388-021-01948-6)
Supplement: Supplementary file 2 — Legends of supplementary figures and Tables [file 41388_2021_1948_MOESM2_ESM.docx]

**Supplementary Figures and Tables Legends**

**Supplement Figure S1** Homology analysis of nucleotide sequence of UBE2C pseudogene and UBE2C gene from human genome.

**Supplement Figure S2** Knockdown of UBE2CP3 inhibits GC cell growth and cell cycle distribution. (A) Knockdown of UBE2CP3 inhibited cell growth rate of GC cell lines. (B, C) Knockdown of UBE2CP3 significantly affected the distribution of different cell cycle phases in GC cell lines. (D) The overexpression efficiency of UBE2CP3 was verified in GC cell lines by qRT-PCR assay. The P values were estimated using one-way ANOVA test. **, P < 0.01.

**Supplement Figure S3** Using different network tools to predict the miRNA that UBE2CP3 can absorb by acting as ceRNA. (A) The miRNAs that UBE2CP3 can absorb were predicted by Annolnc web tool. According to the prediction results, UBE2CP3 contains 3 miR-138-5p binding sites. (B) The miRNAs that UBE2CP3 can absorb were predicted by miRcode web tool. (C) The miRNAs that UBE2CP3 can absorb were predicted by StarBase web tool.

**Supplement Figure S4** The effect of UBE2CP3 overexpression on the expression of ITGA2. (A) The ITGA2 expression was determined by qPCR assay in the MGC803 cell line overexpressing UBE2CP3. The P values were estimated using one-way ANOVA test. **, P < 0.01. (B) The ITGA2 protein level was determined by western blotting assay in the MGC803 cell line overexpressing UBE2CP3.

**Supplement Figure S5** Potential RNA-RNA interaction region between IGFBP7 mRNA and UBE2CP3 mRNA was analyzed by IntaRNA web tool. (A) The predicted RNA-RNA interaction site and folding energy between IGFBP7 mRNA and UBE2CP3 mRNA was shown in the plot. (B) The predicted RNA-RNA interaction site was located at the 3’UTR of IGFBP7.

**Supplement Figure S6** Knockdown of IGFBP7 significantly decreased the expression level of ITGA2 in GC cell lines. (A) The knockdown efficiency of IGFBP7 was verified in the RNA-seq data of SGC7901 cells. (B) The transcripts abundance of ITGA2 detected by the RNA-seq of IGFBP7-depletion was shown in the plot. The ordinate represents counts per million (CPM) value (0-400). (C) The ITGA2 expression level was detected after knockdown of IGFBP7 in different GC cell lines. The P values were estimated using one-way ANOVA test. **, P < 0.01. (D) IGFBP7 expression was upregulated in GC tissues compared to the gastric normal tissues. The P values were estimated using one-way ANOVA test. **, P < 0.01. (E) The overexpression of IGFBP7 predicted a poor prognosis (OVS+DFS) in GC patients from TCGA cohort. The P values were estimated using log-rank test. (F) The overexpression of IGFBP7 predicted a poor prognosis (OVS+DFS) in GC patients from GSE62254 cohort. The P values were estimated using log-rank test.

**Table S1:** The full-length cDNA sequence of UBE2CP3 according to the sanger sequencing.

**Table S2:** The RNA-seq data of UBE2CP3 knockdown in SGC7901 cells.

**Table S3:** The mass spectrometry (MS) analysis of the proteins in specifically differential band around 90 kDa in RNA pulldown assay.

**Table S4:** The transcription factors that bind to the UBE2CP3 promoter are analyzed by Cistrome web tool.

**Table S5:** The information of siRNAs and primers used in this study.

**Table S6:** The information of antibodies used in this study.
