# Supplementary figures and images for: The ELF3-regulated lncRNA UBE2CP3 is over-stabilized by RNA–RNA interactions and drives gastric cancer metastasis via miR-138-5p/ITGA2 axis
Source: Oncogene. 2021 Jul 17;40(35):5403–15. doi: 10.1038/s41388-021-01948-6 (PMC8413130; doi:10.1038/s41388-021-01948-6)

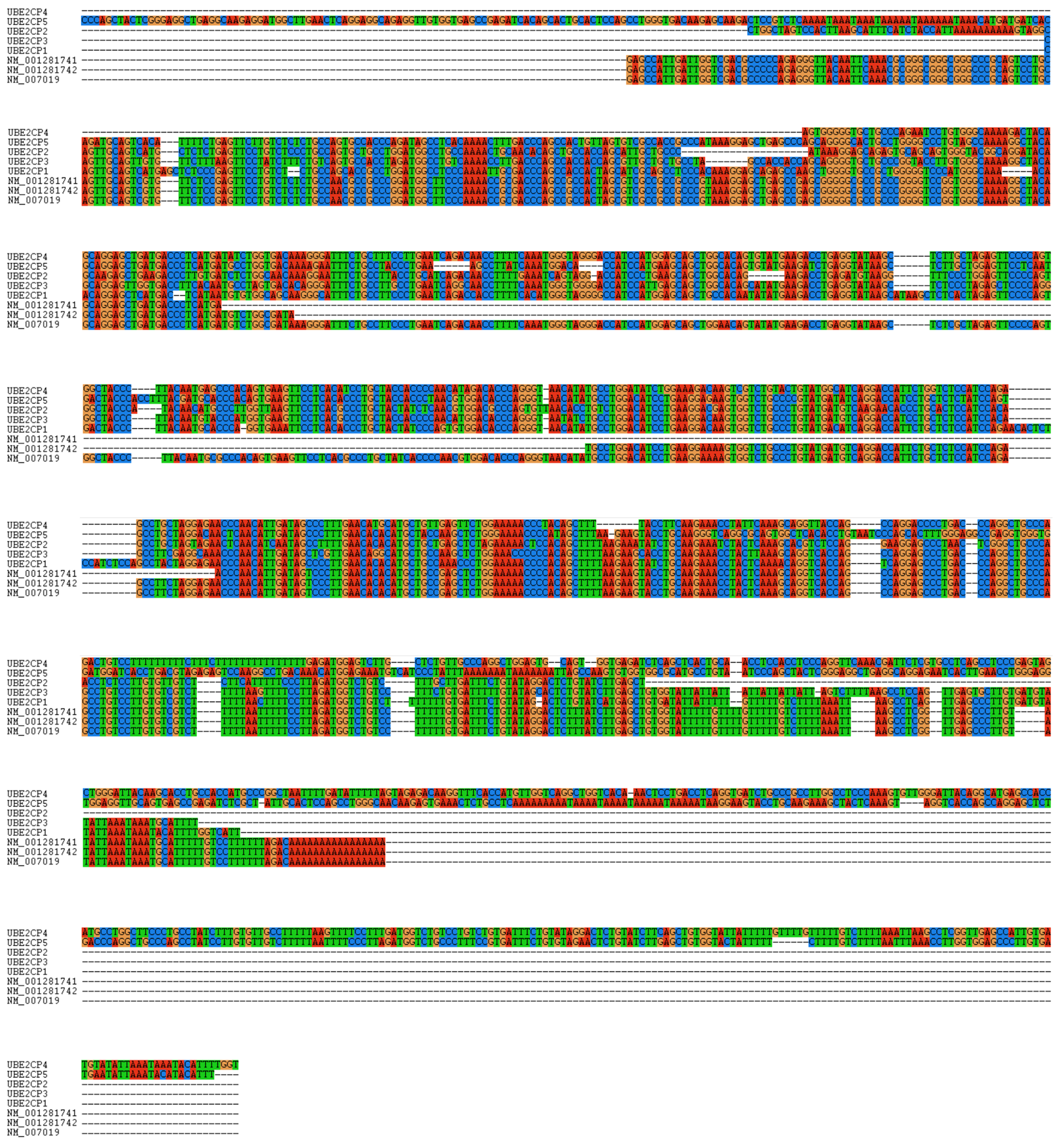

Supplement: Supplementary file 3 — Supplementary Figure S1 [file 41388_2021_1948_MOESM3_ESM.tif]

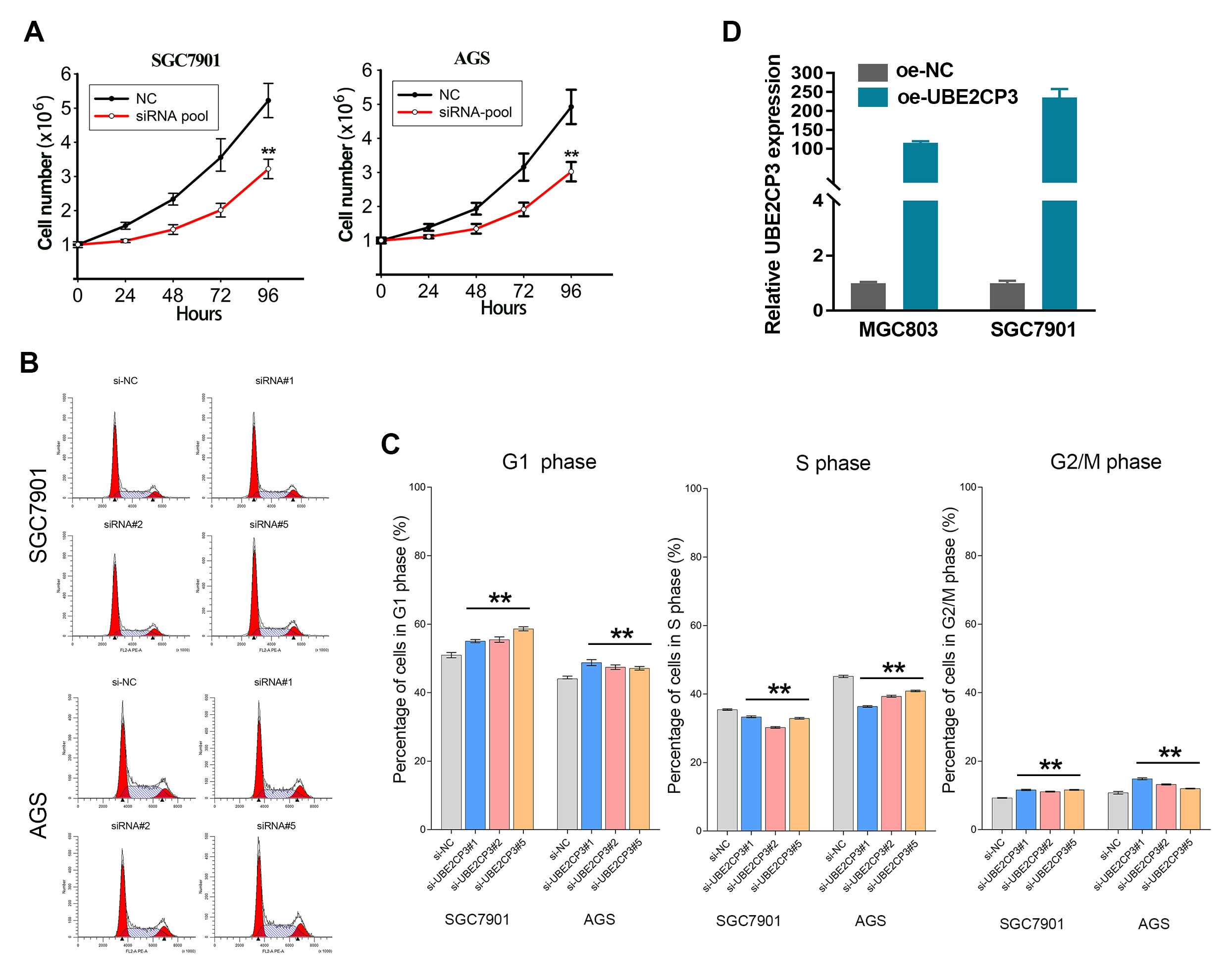

Supplement: Supplementary file 4 — Supplementary Figure S2 [file 41388_2021_1948_MOESM4_ESM.tif]

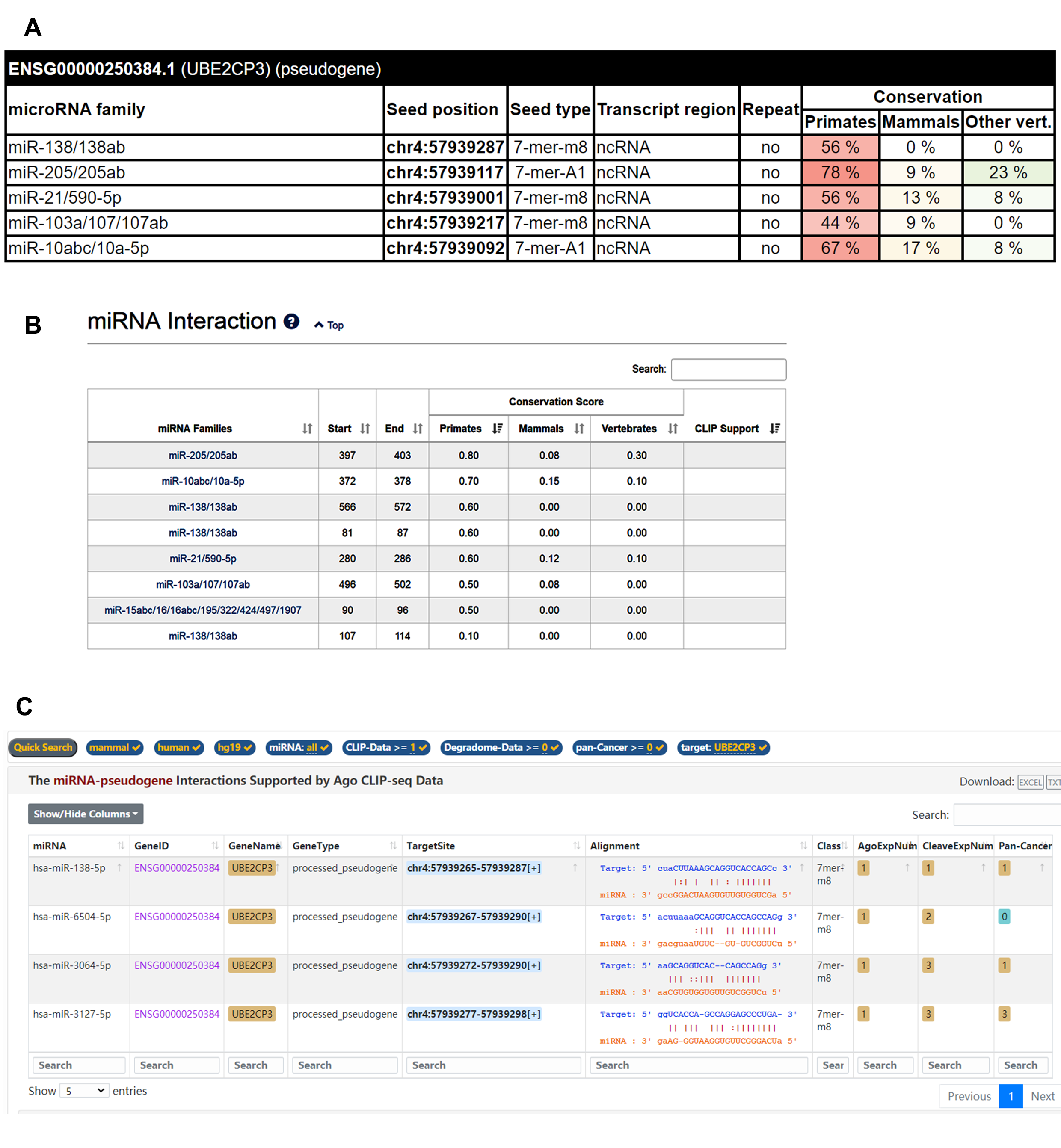

Supplement: Supplementary file 5 — Supplementary Figure S3 [file 41388_2021_1948_MOESM5_ESM.tif]

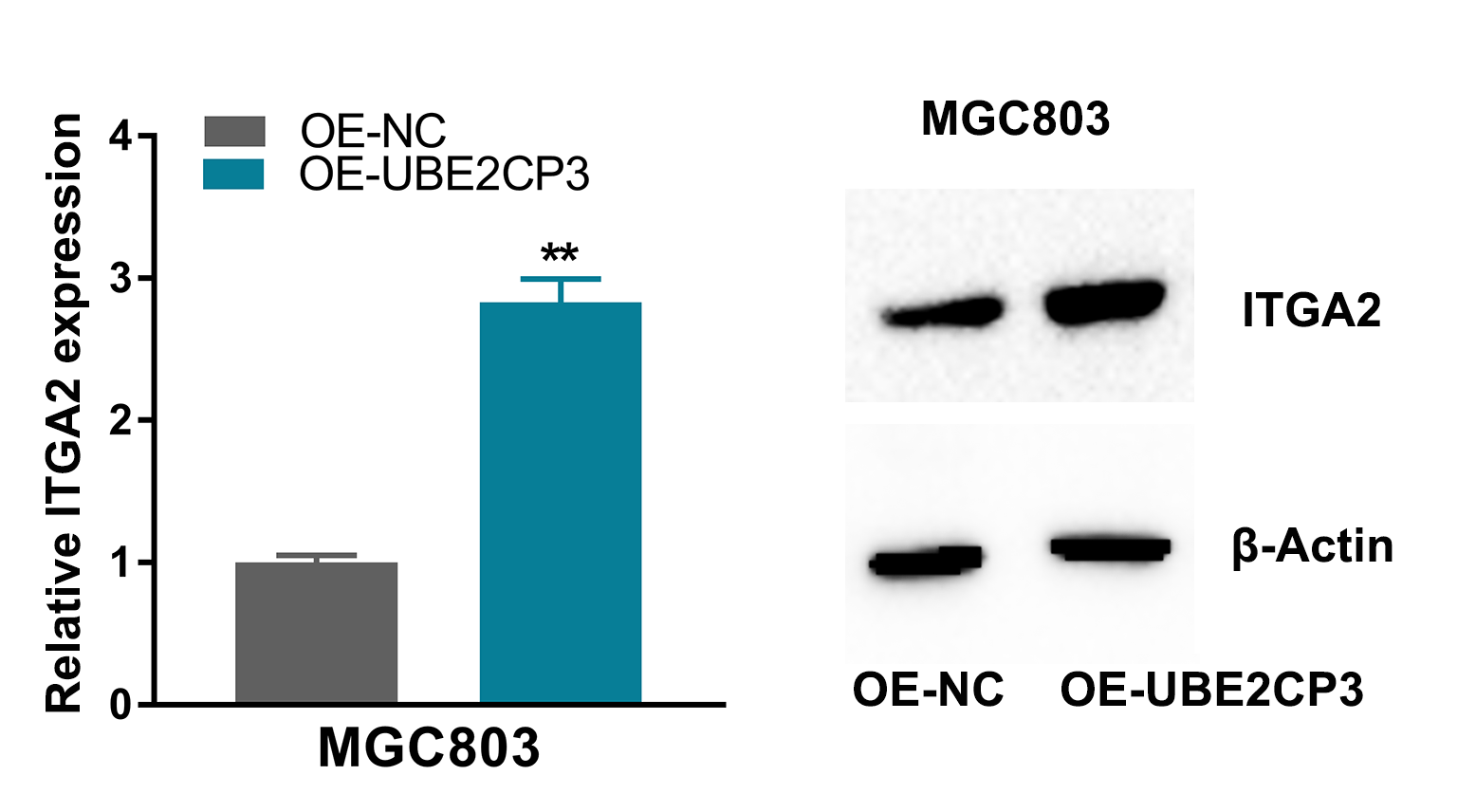

Supplement: Supplementary file 6 — Supplementary Figure S4 [file 41388_2021_1948_MOESM6_ESM.tif]

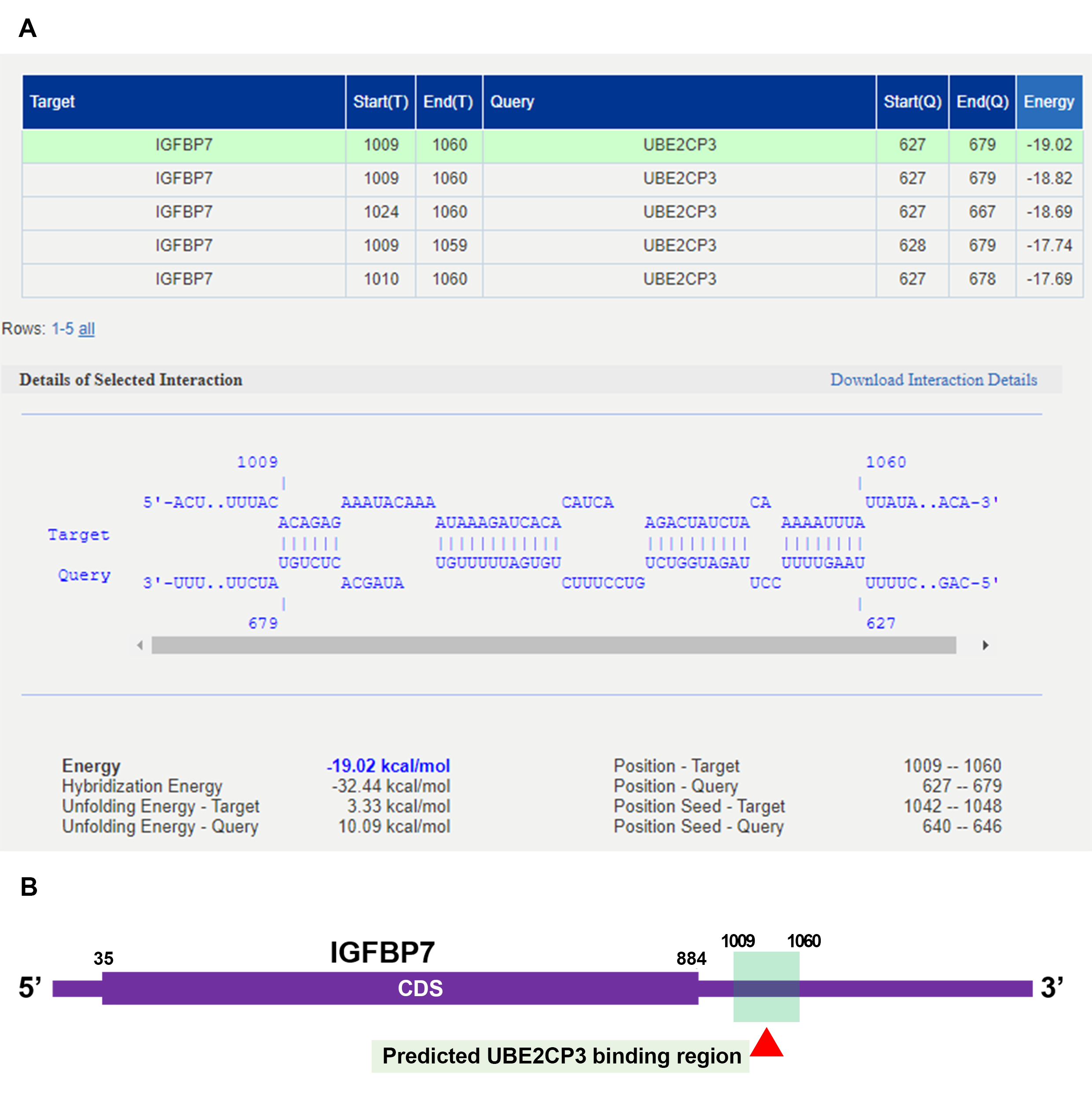

Supplement: Supplementary file 7 — Supplementary Figure S5 [file 41388_2021_1948_MOESM7_ESM.tif]

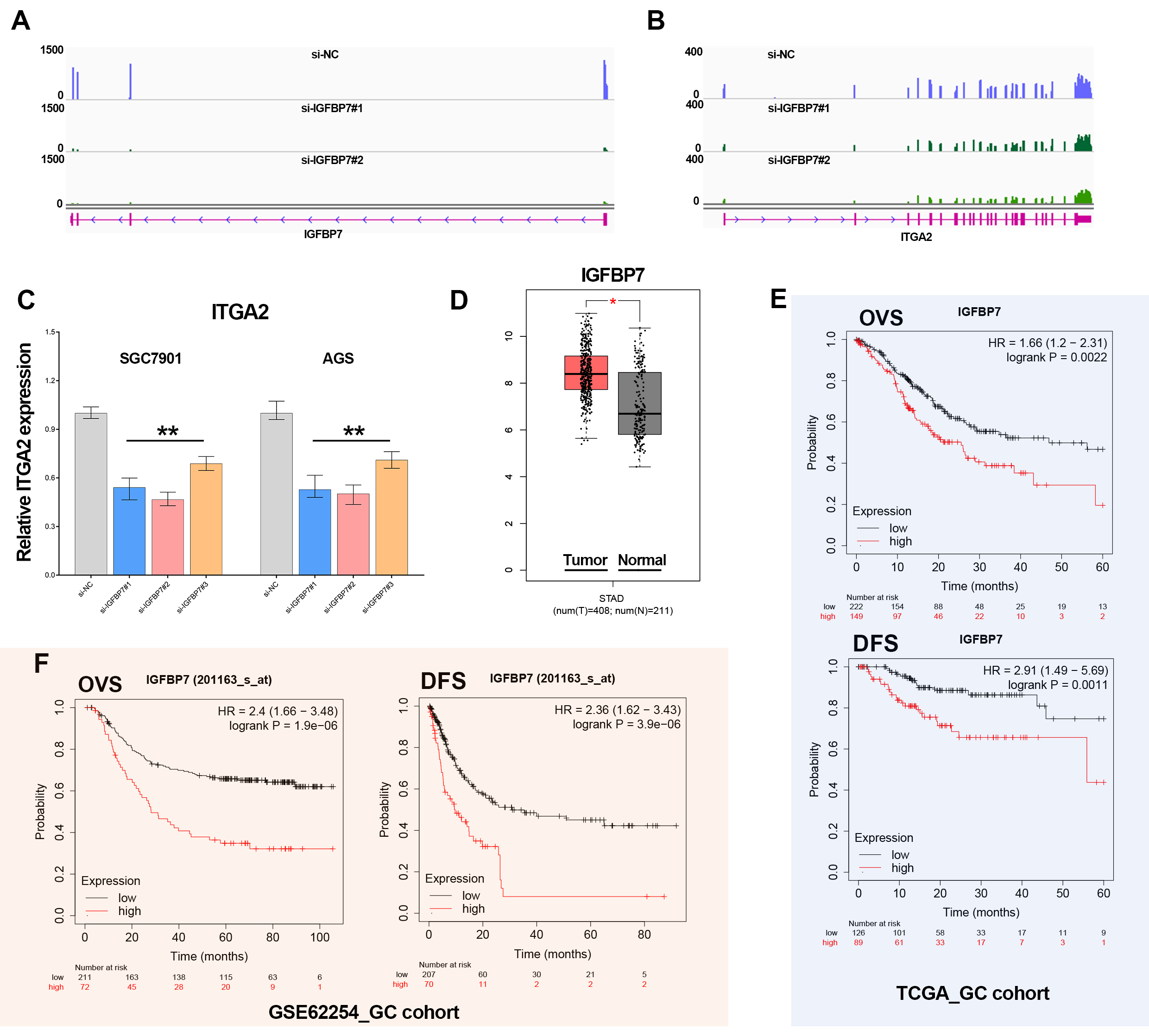

Supplement: Supplementary file 8 — Supplementary Figure S6 [file 41388_2021_1948_MOESM8_ESM.tif]
