# Supplementary material for: The ELF3-regulated lncRNA UBE2CP3 is over-stabilized by RNA–RNA interactions and drives gastric cancer metastasis via miR-138-5p/ITGA2 axis
Source: Oncogene. 2021 Jul 17;40(35):5403–15. doi: 10.1038/s41388-021-01948-6 (PMC8413130; doi:10.1038/s41388-021-01948-6)
Supplement: Supplementary file 9 — Supplementary Table S1 [file 41388_2021_1948_MOESM9_ESM.docx]

**Table S1.**

The sequence of UBE2CP3 gene was verified by Sanger sequencing.

>UBE2CP3

CAGTTGCAGTTGTGTTCTTTAAGTTCCTATCTTTCTGTCAGTGCCACCTAGATGGCCTGTCAAAACCTTGACCCAGCCACCACCAGCGTTGCTGCTGCCTATAAAGGTGCCACCCCCAGCAGGGGTGCTGCCCGGTACCTTGTGGGCAAAAGGCTACAGCAGGAGTTGGTGACCTTCACAATGCCTAGTGACACAGGGATTTCTGCCTTGCCTGAATCAGGCAACCTTTTCAAATGGGTGGGGACCATCCATTGAGCAGCTGGCACAGCATATGAAGACCTGAGGTATAAGCTCTCCCTAGAGCTCCCCAGGGGCTACCCTTACAATGTACCCATGGTGAAGTTCCTCACGCCCTGCTACCACCCCAACATGGACTCCCAGGGTAATATCTGCCTGGACATCCTGAAGGACAAGTGGTCTGCCCTGTATGATGTCAGGACCATCCTGCTCTCCATCCACAGCCTTCGAGGTGAACCCAACATTGATAGCTCGTTGAACAGGCATGCTGCCAAGCTCTGGAAACCCCCCACAGCTTTTAAGAAGCACCTGCAAGAAACCTACTTAAAGCAGGTCACCAGCCAGGAGCCCTGACCCAGGCTGCCCAGCCTGTCCTTGTGTCGTCTTTTTAAGTTTTCCTTAGATGGTCTGTCCTTTCTGTGATTTTTGTATAGCACTCTGTATCTTGAGCTGTGGTATTATTATTATTATTAGTCTTTTAAGCCTCCAGTTGAGTGCTTGTGATGT
