# Supplementary material for: The ELF3-regulated lncRNA UBE2CP3 is over-stabilized by RNA–RNA interactions and drives gastric cancer metastasis via miR-138-5p/ITGA2 axis
Source: Oncogene. 2021 Jul 17;40(35):5403–15. doi: 10.1038/s41388-021-01948-6 (PMC8413130; doi:10.1038/s41388-021-01948-6)
Supplement: Supplementary file 13 — Supplementary Table S5 [file 41388_2021_1948_MOESM13_ESM.docx]

**Table S5.**

Primers and siRNAs used in this study.

| Gene Name | Sequence (5’→3’) | |
| --- | --- | --- |
| The sequence of siRNAs used in this study | | |
| siRNA name | Sense strand | Antisense strand |
| si-NC (Universal) | UUCUCCGAACGUGUCACGUTT | ACGUGACACGUUCGGAGAATT |
| siUBE2CP3#1 | GCCUAGUGACACAGGGAUUTT | AAUCCCUGUGUCACUAGGCTT |
| siUBE2CP3#2 | CCUGAAGGACAAGUGGUCUTT | AGACCACUUGUCCUUCAGGTT |
| siUBE2CP3#3 | GCAAGAAACCUACUUAAAGTT | CUUUAAGUAGGUUUCUUGCTT |
| siUBE2CP3#4 | AAGCCUCCAGUUGAGUGCUTT | AGCACUCAACUGGAGGCUUTT |
| siUBE2CP3#5 | CCACCUAGAUGGCCUGUCATT | UGACAGGCCAUCUAGGUGGTT |
| siELF3-#1 | GCUGCAACCUGUGAGAUUATT | UAAUCUCACAGGUUGCAGCTT |
| siELF3#2 | CCUCUGCAAUUGUGCCCUUTT | AAGGGCACAAUUGCAGAGGTT |
| siELF3#3 | CCAUGAGGUACUACUACAATT | UUGUAGUAGUACCUCAUGGTT |
| si-IGFBP7#1 | GGGUCACUAUGGAGUUCAATT | UUGAACUCCAUAGUGACCCTT |
| si-IGFBP7#2 | UGCUGAUGCUGAAGCCUGUCCUUGG | CCAAGGACAGGCUUCAGCAUCAGCA |
| si-IGFBP7#3 | UAGAGGAGAUACCAGCACCCAGCCA | UGGCUGGGUGCUGGUAUCGCCUCUA |
| si-ITGA2#1 | ACUGGAGGUUUUCUCACAUTT | AUGUGAGAAAACCUCCAGUTT |
| si-ITGA2#2 | GGAGGAGACAACUUUCAGATT | UCUGAAAGUUGUCUCCUCCTT |
| miRNA mimics-NC | UUCUCCGAACGUGUCACGUTT | ACGUGACACGUUCGGAGAATT |
| miR-138-5p mimics | AGCUGGUGUUGUGAAUCAGGCCG | GCCUGAUUCACAACACCAGCUUU |
| Inhibitors-NC | CAGUACUUUUGUGUAGUACAA | |
| miR-138-5p inhibitors | CGGCCUGAUUCACAACACCAGCU | |
| The sequence of RT (reverse transcription) and qPCR (quantification PCR) primers used in this study | | |
|  | Forward primer | Reverse primer |
| UBE2C_qPCR | CCCCAGAGGGTTACAATTCA | GCTCCTGCTGTAGCCTTTTG |
| UBE2CP1_qPCR | CTCCCGAGTTCCTGTCTCTG | CCTTGCTGCCACACATTATG |
| UBE2CP2_qPCR | ACGGCAGGATACAGCAAGAG | AGGCTATTGATGTTGAGTTCTACTAGC |
| UBE2CP3_qPCR | GTTGCTGCTGCCTATAAAGGT | CCTGTGTCACTAGGCATTG |
| UBE2CP4_qPCR | GCTGGGATTACAAGCACCTG | GCTCAACCGAGGCTTAATTT |
| UBE2CP5_qPCR | GTCCAAGGCCTGACAAACAT | TCCTGGCTGGTGACCTACTT |
| ELF3_qPCR | GAAGTGACGTGGACCTGGAT | CTTCTTGCCCTCGAGACAGT |
| ACTIN_qPCR | ATCGTCCACCGCAAATGCTTCTA | AGCCATGCCAATCTCATCTTGTT |
| ITGA2_qPCR | AACAAGCATTCCAAATGTTACTGA | AACTACCATTACTTTCGTAGCACT |
| IGFBP7_qPCR | CACTGGTGCCCAGGTGTACT | TTGGATGCATGGCACTCATAT |
| IGFBP7_RIP_2 | CTGCATGGTTAAAAGTAGTCATGG | TGATATGCATGCTTTTCTTCTG |
| IGFBP7_RIP_1 | TTCCTCCTCTTCGGACACCT | CTCTTCACGCACTCCATGC |
| UBE2CP3_oe_FL | CAGTTGCAGTTGTGTTCTTTAA | ACATCACAAGCACTCAACTGGAG |
| miR-138_RT | GTCGTATCCAGTGCGTGTCGTGGAGTCGGCAATTGCACTGGATACGACCGGCCTG | |
| miR-138_qPCR | GGGAGCTGGTGTTGTGAATC | CAGTGCGTGTCGTGGAGT |
| U6_ qPCR | CTCGCTTCGGCAGCACA | AACGCTTCACGAATTTGCGT |
| U6_RT | AACGCTTCACGAATTTGCGT | |
| The sequence of chip-qPCR primers used in this study | | |
|  | Forward primer | Reverse primer |
| UBE2CP3_P | GGCCTATTTGGGGCTTTTAC | GGCCACATGTGTGCTGTTAG |
| Ubiquitin_P | TGGGTCCGATTATTGAATGG | AGCTGGGTGTCCAGGTTAAA |
