# Supplementary material for: The ELF3-regulated lncRNA UBE2CP3 is over-stabilized by RNA–RNA interactions and drives gastric cancer metastasis via miR-138-5p/ITGA2 axis
Source: Oncogene. 2021 Jul 17;40(35):5403–15. doi: 10.1038/s41388-021-01948-6 (PMC8413130; doi:10.1038/s41388-021-01948-6)
Supplement: Supplementary file 14 — Supplementary Table S6 [file 41388_2021_1948_MOESM14_ESM.docx]

**Table S6.**

The information of antibodies used in this study.

| Protein Name | Brand | Catalog NO. | Dilution ratio |
| --- | --- | --- | --- |
| GAPDH | Proteintech | 60004-1-Ig | 1:5000 |
| β-Actin | Abbkine | A01011 | 1:10000 |
| Vim | Proteintech | 10366-1-AP | 1:3000 |
| CDH1 | Proteintech | 20874-1-AP | 1:4000 |
| EPCAM | Proteintech | 21050-1-AP | 1:1500 |
| ZEB1 | Proteintech | 21544-1-AP | 1:1000 |
| ITGA2 | ABclonal | A19068 | 1:2000 |
| ELF3 | Santa Cruz | sc-376055 | 1:1000 |
| ILF3 | Proteintech | 19887-1-AP | 1:5000 |
